# Spirit of the Foreign Periodicals, &c.

**Published:** 1838-07-01

**Authors:** 


### Spirit of the Foreign Periodicals, &c.

### Occasional Existence of Animalculæ in the Human Blood.

The occasional existence of Animalculæ in the human blood has been admitted, for a long time, by some of the best authorities in Physiology.

Brera and Treutler were the first to discover and announce the presence of the *polistoma* (one species of these entozoa) in venous blood. M. delle Chiaje, who has long devoted himself to microscopic researches on this subject, has confirmed the accuracy of these gentlemen's statements, and he has also minutely prosecuted his enquiries respecting the physical characters of the animalculæ, and the mode of their generation.

He is inclined to regard the *polistoma* as a result or product of a pathological condition, and, therefore, of a spontaneous development or generation—as well indeed as all other *entozoa*. The morbid products, he says, may become organised and live at first at the expense of the tissue, in which they arise; they may then detach themselves from these, and in this state enjoy an independent existence—as for example the *Acephalocysts* or *Hydatids*. The most simple *Acephalocysts*, (those of which the animal character was so long called in question), are now admitted to be genuine animalculæ, provided with a head, and also with special suckers, according to the researches of M. delle Chiaje.

With respect to the *Polistoma*, M. Chiaje informs us that he has discovered worms of this genus in two cases only hitherto. Both patients were labouring under phthisical disease.

In one, the blood rejected during the first attack of hæmoptysis, exhibited, half-an-hour afterwards, several small flat worms, somewhat similar to minute leeches, and either floating in the serum, or adhering to the sides of the vessel—which had been perfectly clean, before the blood was received into it.

The two physicians in attendance showed them to M. delle Chiaje, who at once recognised them to be the *Polistoma Sanguinea*. He examined them minutely with the aid of a microscope, and quite satisfied himself of their real character. The same phenomena were observable in the subsequent hæmoptoic attacks of this patient.

The second case was very similar to the one now mentioned. A young man was labouring under hæmoptysis: the rejected blood, on several occasions, presented a considerable number of these worms to sight.

M. Chiaje alludes to the case detailed by Mr. Bushman of Dumfries, and recorded in the number of the "Medico-Chirurgical Review," for January 1834. (It may be interesting to reproduce a brief account of this case here.

The patient, a young boy, was labouring under *influenza*; for which he was bled. In the blood, after it had remained at rest for an hour, Mr. B. discovered numerous worms, some floating in the serum, and others imbedded in the crassamentum.

Mr. Rhind of Edinburgh, an able naturalist, drew up an accurate report of these animalculæ. They were from one half to two-thirds of an inch long; they consisted of an articulated body, a head with rudiments of antennæ and palpi, and a tail terminating in two tubular bodies, or stigmata. The colour of the animals were bright red. In structure, colour, and size they correspond, says Mr. Rhind, exactly to the larvæ of the *Tipula oleracea* fly, which in Summer is so abundantly found in ditch and river water.)

Various other cases also are mentioned. Thus Notorianni has informed us that he discovered thirteen worms of the genus *Polistoma* in one of the sinuses of the aorta; and Borelli, Redi, Vallisnieri, and Lucarelli have recorded similar instances.

M. Chiaje has extended his enquiries to the blood of the lower animals; and he has discovered that animalculæ are often present in the circulating fluids, not only of the higher Vertebrata, but also of many of the Invertebrata. Thus, in the blood of several Sepiæ and also of the Sea-mouse, he has succeeded in detecting occasionally a species of round or ovular animalcule. The mesenteric vessels of the *Rana pipa*, and the cranial veins of the porpoise have been found to contain worms similar to the *Polistoma*. We also know that the Aneurismal sacs of the Meseraic vessels in the horse frequently contain the *Strongylus Armatus;* and that M. Andral has met with *Acephalocysts* in the pulmonary veins of man.—(*Clinique Medic.* t. iii.) *Annali Universali di Medicina.*

---

REMARKS ON CONSTITUTIONAL HÆMORRHAGES WITH CASES. USE OF THE SULPHATE OF SODA AS AN INTERNAL REMEDY.

*Case* 1. A man, twenty-four years of age, was admitted into the Hôtel Dieu, in consequence of a false aneurismatic swelling in the palm of the right-hand, which, having been mistaken for an abscess, had been opened. This man had been subject, since his boyhood, to frequent recurrence of Epistaxis, and to very profuse bleedings from any accidental wounds.

His life had once been seriously endangered by the hæmorrhage from some leech-bites on one of his knees. The swelling of the hand had come on after a violent strain, which, he (the patient) said, had dislocated his thumb-joint at the time. The surgeon, who first saw it, mistook it for an abscess, in consequence of the absence of all pulsation and from the distinct sense of fluctuation, which was perceptible in it. The hæmorrhage had been very abundant, and could not be restrained by pressure on the radial and ulnar arteries, but only by firm pressure on the wound itself. Whenever the compresses were removed, it returned; and M. Roux, therefore, determined at once to tie the arteries of the fore-arm. The radial one was first secured; and, as the bleeding still continued, the ulnar also was tied. The hæmorrhage seemed to be arrested for the time; but, in less than an hour, all the bandages were found soaked with blood, which had oozed not only from the palm of the hand, but also from the two incisions in the fore-arm, which had been made during the operations. Suspecting that the blood might continue to flow through the interosseous arteries, the dresser applied a tourniquet above the elbow-joint; but even this failed, and the patient died in the course of the evening.

The dissection was performed with great care. Almost all the viscera of the three great cavities were pale and exsanguine: some fluid blood was found in the right, but none in the left, cavities of the heart. The blood-vessels of the affected limb were minutely injected; but no irregularity, either as to distribution or visible formation of their branches, was anywhere discoverable. The radial and ulnar arteries had been fairly tied; there were, however, no traces of any coagulum either above or below the seats of the ligatures. The deep palmar arterial arch was *intact*—(in other words, was not involved in the aneurismatic tumor). The anastomosing branch, which passes between the superficial and deep arches, appeared to be the only one which was lost in the aneurismatic sac. The soft parts surrounding and forming this sac were reduced to a state of a blackish *bouillie*, in which no distinction of tissues could be discovered.

The reporter of the preceding case is inclined to attribute the diseased action, —which, we may suppose, gave rise to the very remarkable disposition to hæmorrhage,—rather to an extreme fluidity of the blood itself, than to any change of structure in the blood-vessels, or in any other of the solid parts. That the blood was, indeed, remarkably fluid, and uncoagulable, cannot be disputed; but as to the cause of this change we are quite in the dark.

*Case* 2. A man, forty-one years of age, who had frequently suffered from profute *epistaxis* and *hœmaturia*, and also from rheumatic pains, accidentally struck his side against the edge of a door: the consequence of this was an enormous ecchymosed swelling, accompanied with extreme general languor and tendency to syncope.

On a former occasion, the mere pressure of another person's arm on the patient's elbow had caused a most extensive extravasation of blood from that part up to the shoulder.

It is worthy of notice that two uncles of this man had died, when young, from accidental hæmorrhages: one of them from the bleeding after the extraction of a tooth.

His only sister too died in her infancy from a hæmorrhage from the vulva; and his two brothers, after having been subject for many years to violent epistaxis, perished; the one in consequence of a blow on the head, which had caused an enormous sanguineous infiltration of the scalp, and the other from secondary hæmorrhage after ligature of the crural artery, rendered necessary by the profuse bleeding from an accidental wound in the calf of the leg. (*Revue Medicale*, October, 1835.)

The *Hæmorrhagic Diathesis*, or *Constitution* seems to have been very remarkable in this family.

*Case* 3. In the number of the *Archives Generales* for October 1833, there is the detailed history of a case of hæmorrhagic diathesis, in which, as in the preceding one, the patient, a young boy, was afflicted at the same time with rheumatic pains. Dr. Hugues had made diligent enquiry as to the health of the other members of his family, and had learned the following particulars. All the *males* were subject to occasional spitting and vomiting of blood; also to hæmaturia, epistaxis, and to troublesome hæmorrhage from any part, which chanced to be wounded. All of them suffered from troublesome rheumatic pains, whenever they were affected with any form of hæmorrhage. Several had died in their youth; and in those, who survived to more mature years, the hæmorrhagic disposition seemed to become less and less decided. It had been frequently remarked that, if any of them chanced to be blistered, the discharge from the vesicated surface was generally bloody.

Several of the *females* of this family, although they have exhibited the hæmorrhagic diathesis in their own case, had transmitted it, *sans exception!* to their offspring.

If the preceding observations are quite accurate and to be depended upon, they present a very remarkable instance of a peculiarity of constitution in the *males* of a family, while the *females* were exempt from it. The co-existence of the hæmorrhagic attacks and of rheumatic pains,—the former always preceding the latter—deserves also to be noticed.

In the Archives for July 1835, we find some particulars of a family, the *males* of which exhibited a similar disposition to troublesome hæmorrhages. One, a boy nine years of age, perished from excessive bleeding after the application of cupping-glasses to the knee; another, three years younger, died from a wound on one of his temples, from which there had been a most profuse hæmorrhage; a third, thirteen years of age, nearly lost his life from the obstinate bleeding from leech-bites upon his shoulder.

Two of these children had long suffered from rheumatic pains.

*Case* 4. A man, forty years of age, and of a soft lymphatic constitution, was subject to most troublesome hæmorrhage from very slight wounds. On two occasions the bleeding after the extraction of a tooth had required, for its arrest, continued compression for several days. At another time it was necessary to use the actual cautery to stop the bleeding from leech-bites; and the same means were, on one occasion, requisite to stop the bleeding from a simple wound of a finger. Several times large ecchymoses had formed after very slight bruises.

It was for a swelling of this sort on the inner side of the thigh, that he had consulted M. Marjolin. This eminent surgeon was inclined to attribute the singular tendency to hæmorrhage, which existed in such a case as the present, rather to an atony of the capillary vessels, than to any marked fluidity of the blood itself, although he was not disposed to deny some influence to this latter cause.

It deserves to be noticed, in reference to this subject, that wounds of the larger blood-vessels, such as that made in venæsection, are usually found to heal as readily in *hæmorrhagic* individuals as in any other set of patients. This, however, is not always the case. Dr. Otto of Philadelphia, has recorded the history of a woman, *all of whose sons* had a remarkable tendency to troublesome hæmorrhages from the slightest wounds. One, if not two, of these sons died in consequence of the loss of blood from ordinary venæsection. A curious circumstance is mentioned of the extraordinary power of the *sulphate of soda* in arresting hæmorrhage from some of those persons. It was found that if this salt were taken, for several days successively, in doses large enough to purge the bowels, the bleeding ceased, and the wound speedily began to cicatrise.

Krimer also alludes to the efficacy of this remedy in such cases. He alludes particularly to the history of one family, of which all the *male descendants, for four generations,* had been most strikingly subject to hæmorrhagic accidents.

M. Sanson, in his Concours thesis for 1836, mentions the case of a man, who died from a urethral hæmorrhage: six of his children had perished from the bleeding from casual wounds.

Mr. Blagden has given us the particulars of a man, who nearly perished from hæmorrhage after the extraction of a tooth: it was not completely checked for nearly three weeks.

This patient subsequently had an alarming hæmorrhage from a trifling wound of the scalp; and, on a future occasion, the bleeding which followed the extraction of a tooth proved so obstinate as to require the ligature of the carotid artery, before it was arrested! The particulars of this case are reported in Samuel Cooper's "Dictionary of Surgery."

In concluding these remarks on Constitutional hæmorrhage, we may once more state that the cause of this most serious condition may be either an atony and defect of contractility in the minute blood-vessels of the part, or a dissolved and unusually thin state of the blood itself, which is thus but little disposed to coagulation. Probably both of these agencies are present in most cases.

Finally, in the treatment of such cases, the use of internal astringents has, perhaps, been hitherto too much neglected. The use of the preparations of lead and of some other mineral astringents may probably contribute very materially to promote the utility of local styptic applications. We have found it stated in the report of one of the preceding cases that the administration of large doses of sulphate of soda had a marked effect in arresting the hæmorrhage. Was this owing at all to the rapid discharge of serum by copious watery stools?

When we have reason to suspect any scorbutic tendency in the patient's constitution, the use of lemon-juice and of other vegetable and mineral acids, should, as a matter of course, be adopted.—*Archives Generales.*

---

### Remarks on Spasm of the Glottis. Influence of Enlargements of the Thymus Gland.

This affection has, it is well known, attracted great attention since the publication of Dr. Ley's elaborate work on *Laryngismus Stridulus.* Among the causes of this obscure malady, there is one which hitherto has been much overlooked in this country, (Britain,) but which has been repeatedly insisted upon of late years in Germany—we allude to an enlarged or hypertrophied state of the thymus gland.

This form of the disease is in truth the "*asthma thymicum*" of continental writers. Before Dr. Ley propounded his ingenious speculations as to the influence or *modus operandi* of swellings of the cervical glands in inducing spasmodic affections of the larynx, it was supposed that the mere pressure of the enlarged thymus gland acted directly by contracting the passage of the air-pipe.

The difficulty however of explaining a paroxysmal disease by reference to a permanent organic alteration was always felt; and we therefore applaud Dr. Kyll, the author of a very excellent paper on spasm of the glottis in a recent number of Rust's Magazine, when he attempts to shew that enlargements of the thymus gland operate rather *indirectly* by pressure on some of the laryngeal nerves, than *directly* by contracting the passage of the larynx itself. We are so entirely ignorant of the functions and uses of this gland, that we cannot indeed hope to arrive at any very satisfactory views of its pathology. Whether enlargements of its structure may act in other ways, besides that of mere mechanical pressure, in inducing laryngeal and some cephalic affections, we cannot say; but of this we are assured from the results of our own experience, that in not a few cases of convulsions and of rapidly fatal hydrocephalus* in infants, the thymus is found extremely hypertrophied, and occasionally charged with an unusual quantity of milky serum throughout its tissue.

We may, *en passant,* allude to the frequent co-existence of lesions of the heart, with hypertrophy of the thymus gland—a complication, which will account for the extreme tendency of children so affected to convulsive disease of all forms.

The following case of excessive *hypertrophy of the thymus* will be read with interest.

A fine healthy infant, six months old, awoke most unexpectedly from a long sleep, with all the symptoms of spasm of the glottis: these continued for two or three minutes and then ceased, so that he fell asleep again, and was as well as usual next morning.

A similar paroxysm returned for many nights successively; and, in the course of a short time, these attacks became more frequent and of longer duration, and the child was occasionally seized with them during the day, more especially after laughing, crying, &c. &c. The disease was attributed to teething Two months after the first attack, Dr. Kyll was summoned one night, in consequence of an unusually severe paroxysm, followed by general convulsions, which lasted for upwards of an hour. The head was hot, and the carotids were felt beating with great violence. The symptoms never fairly gave way, or yielded to the remedies employed; and the child died on the 10th or 11th day after this last severe paroxysm.

On dissection, the thymus gland was found enormously enlarged; it covered all the anterior part of the chest, and extended from the larynx downwards as low as the diaphragm. The lungs and pericardium were quite concealed by it, and it firmly compressed the jugular veins at their termination: its weight was upwards of 13 drachms. The jugular veins in the neck were manifestly dilated. The right cavities of the heart were soft and enlarged; and the left ventricle, on the contrary, was thicker and firmer than usual. The vessels of the brain were gorged with dark blood, and the ventricles contained a quantity of serum.

It is quite evident that the hypertrophy of the thymus was the primary disease in the present instance, and that the convulsive and hydrocephalic symptoms were attributable to it.

We have already said that spasm of the glottis may be owing to several very different causes—such as inflammation of the encephalon, or some other form of cerebral irritation; inflammation of the medulla oblongata; dentition; a tumefied state of the cervical lymphatic glands; hypertrophy of the thymus gland, &c.

As a matter of course, each variety of the disease has certain peculiar or diagnostic symptoms, which may assist the cautious physician in determining the genuine character of the case.

---

* Whether the enlarged gland acts, in this case, by compressing the jugular veins, and thus retarding the free return of the blood from the head, is doubtful. Certain it is that many forms of dropsy of the thorax, and more especially of the abdomen, are referrible to such a cause—venous compression.

. Thus in the *cerebral,* and also in the *medullar* variety of the affection, there are always some precursory signs, which will indicate the existence of irritation at some point of the nervous centres : for example, there is a feverish heat of the head, with quickened pulse, general restlessness, tendency to convulsions, irritability of the stomach, and, in one of the forms, tenderness perhaps of some point of the spine.

When the disease is connected with an enlarged state of the glands of the neck, the child will be usually found to be of a scrofulous temperament, weak, subject to cough, and derangements of the stomach and bowels, &c.

It may now be asked, if there are any symptoms which indicate an hypertrophied state of the thymus gland. Fingerhuth is of opinion that auscultation affords considerable assistance in the diagnosis of this affection. He says that, when this gland is much enlarged, the extent and force of the respiratory murmur over the upper and fore part of the chest are considerably impaired, but we feel convinced that not much value can be attached to this sign ; for, independently of the extreme uncertainty which attends all auscultatory explorations in young children, the diminution of the respiratory murmur over the space indicated may be discovered in many cases, where there is not the slightest symptom of disturbed health. Some writers have alleged that enlargements of the thymus gland are usually accompanied with a displacement of the heart *dextrad,* and therefore that its pulsations are to be perceived more towards the right side than in health. But this sign also is utterly valueless. Perhaps more assistance in the diagnosis of the case may be derived from attention to the following circumstances. The children, affected with hypertrophy of the thymus, are usually subject to a dyspnœa more or less permanent; the attacks are more frequent when they are lying on their back ; the tongue is often observed to be protruded from the mouth, even during sleep ; and percussion over the *thymic* region yields a dull sound, similar to that elicited by percussion over the region of the liver.

Whatever be the cause of spasm of the glottis, our *prognosis* should always be very guarded ; for a fatal termination may most unexpectedly occur at a time, when the child seems to be in perfect health. If the spasm continues beyond a minute or two at the utmost, life will be extinguished, whatever the cause of the spasm may have been.

We do not propose at present to make any remarks on the treatment of this very interesting disease, as we should thereby far exceed the limits of this notice. It must be obvious that the treatment must vary very much, according to the cause of the disease, the temperament of the patient, and so forth.

To those, who may wish to pursue the literary history of this affection, the following catalogue may be useful.

Kopp, denkwurdigkeiten in der arztlichen praxis. 3 vol. 1836. Francfort.

Marsh, Dublin Hospital Reports, Vol. 5.

Caspari and Pagenstecher, beschreibung des asthma thymicum. Heidelberger Klin. Annal. Vol. 7.

Schneider, ueber asthma thymicum. Med. Conversations-blatt. No. 46. 1830.

Brueck, ueber Asthma Thymicum. Ib. No. 22. 1832.

Pittschaft, ueber Asthma Thymicum. Ib.

Conradi, ueber Asthma Thymicum, in den Gottinger Anzeigen, No. 32, 1832.

Conradi, handbuch der Speciellen Pathologie und Therapie. Cassel, 1833.

Wanderlich, ueber Asthma Thymicum, in Med. Correspond-blatt. des Wurtemburg Vereins. 1832.

Brunn, ueber Asthma Thymicum, in Wochenschrift fur die ges. heilkunde. No. 49. 1833.

Kornmaul, Inaugural-abhandlung ueber das Asthma Thymicum. Zweibrucken, 1834.

Hirsh, ueber Asthma Thymicum, in Hufeland Journal. Juli, 1835.

Fingerhuth, bemerkungen ueber Hypertrophie des Thymus: in Wochenschrift von Casper. No. 36. 1835.

Rosch zu Schwenningen, ueber Asthma Thymicum : in journal von Hufeland. 1836.

Haugsted, descriptio thymi, &c. Hafniæ, 1832.
Graf, ueber Asthma Thymicum; in jahrbucher des Arztl. Vereins zu Munchen. 11 Jahrgang.
Albers, beobachtungen auf dem gebiete der Pathologie. Bonn. 1836.
*Rust Magazin fur die gesammte heilkunde, von Casper.*

---

### On the Treatment of Phthisis.

The following is the formula of treatment recommended by M. Sue, the chief physician of the Hôtel Dieu at Marseilles, in a late memoir communicated to the Royal Academy of Medicine at Paris, and which has been reported very favourably upon by its commission.

"1. General and topical bloodletting—the cupping-glasses to be preferred to leeches as a local means of depletion—according to the strength of the patient, and the seat and complications of the disorder.

2. The administration of a mild emetic, morning and evening: a grain of tartrate of antimony with a few grains of ipecacuan will be found convenient.

3. The use of raw slugs, as an article of diet, or of ass's milk, according as either of these articles agrees with the patient's system.

4. Frictions on the chest with croton oil, or with antimonial ointment; and, as the disease advances and the tubercles begin to soften, the establishment of several small caustic issues, which should be allowed to suppurate naturally by covering them with simple sparadrap. These tissues should be renewed as the former ones dry up.

5. The application in the evening of the *ioduretted pommade,* (from half a drachm to two drachms,) under each axilla.

6. A syrup, to which M. Sue gives the name of *digitaline,* and which is prepared with digitalis, ʒj., white poppies, ℥j., and cyanuret of potassium, eight grains.

7. The diet to consist of vegetable creams, rice-water and other 'potages maigres,' boiled fruits, eggs, fish, and white meats, &c."

*Remarks.* We (the Rev.) have no objections to offer to the above course of medication in phthisical disease, as it seems to us to be sufficiently rational, and to agree very much with what is usually prescribed by most scientific physicians.

But what will our readers think of M. Sue's discretion, when they are told that he seriously proposes to induce atrophy of a diseased lung, (when there is reason to believe that the disease is confined to one side of the thorax,) by either making and maintaining an opening into the chest, or by tying the bronchus of that side, or, lastly, by keeping up a firm and uniform pressure by means of straps and bandages.—*Rev.*

---

### Two Cases of genuine Gangrene of the Lungs.

*Case* 1. An aged man, deformed and much given to drink, had for many years been subject to a cough, which distressed him greatly during the Winter months. In March 1837, he had, during several days, attacks of vomiting of blood—these, as well as the cough, subsided under the use of nitre and of bitter salt. After a debauch, he was again seized with the *hæmoptysis,* which would not yield to any remedies employed. On practising auscultation of the chest, the sound of pectoriloquy was heard over the gibbous part of the thorax; and the respiratory murmur in other parts was feeble, and blended here and there with a mucous râle. The sputa, which were abundant, were mixed with blood. A diarrhœa came on; and this resisted every astringent. The pulse however kept tolerably well up; and the mental faculties remained clear and unaffected. The breathing only became more embarrassed, &c.; and the patient, on rising out of bed, fell back and expired.

*Dissection.* Both sides of the pleural bag contained a quantity of a yellowish green serosity. The right lung was dark-coloured, flabby, and seemingly exsanguine; the left one was much shrivelled up and reduced in volume; its parenchyma, when cut across, was found to be reduced into a soft, pultaceous mass, of a blackish red colour, and emitting a most offensively fœtid smell. In short, it exhibited all the characters of the *diffused gangrene* of Laennec, in a very advanced degree. There were neither tubercles, hepatization, nor adhesion in this or in the other lung.

The heart was greatly hypertrophied, and of a very flabby texture; the blood in its right cavities was exceedingly thin and serous; and the inner surface of all its cavities, as well as that of the large bloodvessels, was stained with a deep red colour. The parietes of the right ventricle were almost entirely converted into a fatty matter.

*Remarks.* The preceding case (whose details we have much abridged) illustrates very well the extreme uncertainty of diagnosis in some cases. Here there was not only an extensive disorganization of the most vital organs, but a most depraved and corrupted state of the very stream of life, the blood, and yet there were no very alarming symptoms:—the worst were the dyspnœa and looseness of the bowels. The pulse retained its power, and the mind all its clearness to the last!

*Case* 2. The patient had, for a number of years, been subject to cough with expectoration, but with scarcely any difficulty of breathing. He had led a very dissipated debauched life, and still exhibited some traces of syphilitic taint.

Eight days before Dr. Rambold saw him, he had been seized with shivering, dyspnœa, and a very troublesome cough. The features had assumed a pale livid aspect. He did not however complain of any pain in the chest; but there was a sharp uneasiness under the false ribs on either side. The pulse was small, contracted, and quickened; the respiratory murmur was obscure, irregular, and here and there puerile: œgophony was audible over the posterior and middle part of the thorax.

The case was supposed to be one of pleuritic effusion, and of partial pulmonary exudation. As there were no inflammatory or typhoid symptoms, Dr. R. treated the case with a mixture of tartrate of potash, muriate of ammonia and tartrate of antimony—a compound which our author highly recommends in such cases.

During the course of the night, the patient rose several times to go to stool; and in one of these efforts, he suddenly and most unexpectedly expired.

*Dissection.* The left lung was found adhering firmly to the costal pleura. On breaking the adhesions, the pulmonary parenchyma was slightly torn; and from this point there flowed a quantity of thick, viscid, black and fœtid blood. On making an incision there, an excavation, of the size of an egg, was discovered. Into this, several of the bronchi opened.

The greater part of the right lung was hepatised and quite impermeable to air. On cutting into it, there flowed out a mucus-looking fluid, viscid, very fœtid, and of a brownish colour—similar to what the author has seen in other cases of gangrene of the lungs. In the substance of this (the right) lung, there were several spots or patches, of a greenish-black hue, and distinctly gangrenous.

The heart was very small and flabby, and contained some thin dissolved blood.

*General Remarks.* All the cases of pulmonary gangrene may be attributed to one of two causes, either acute inflammation, or a dissolved unhealthy state of the blood—the result very frequently of a dissipated debauched life. M. Rambold is of opinion that most of the cases occur in the course of typhoid diseases. Laennec too seems inclined to the same view; as he considered gangrene of the lung rather as idiopathic, like anthrax or hospital gangrene, than as the effect of a previous active inflammatory action. It has however been distinctly found that this morbid change does occasionally succeed to inflammation of the organ; although, at other times, it seems to take place quite independently of it. Andral has given some examples of the first of these forms in his Clinical Medicine. The colour of the gangrenous part is a greenish-brown, or a dark reddish-black; and its consistence is usually soft and pultaceous. The putrid odour however is perhaps the least uncertain mark of its existence.

M. Chomel has described a gangrenous condition of the lung, which he has found occasionally in the bodies of those, who have died from the effects of exposure to the effluvia from cess-pools and sewers. The lung was black or greenish, full of a sanious and very fetid fluid, and softened and partially deliquescent.—*Medicinische Annalen.*

### Quinine in Enlargements of the Spleen.

There was lately, in the service of M. Andral, at La Charité, a case which illustrated the efficacy of quinine in enlargements of the spleen.

The patient was a shoe-maker, who was in a poor sickly state, in consequence of repeated attacks of ague. The spleen had become enormously engorged; and it was chiefly with the view of obtaining relief from the distress which this occasioned, that this man had gone into the hospital five months ago.

M. Andral had given a very fair trial to the sulphate of quinine in large doses for a length of time—from 12 to 30 grains daily. But no benefit had resulted from this practice.

Subsequently the splenic region was repeatedly blistered ; but with no better effects. Symptoms of ascites have come on, and the strength of the patient has become more and more reduced.

The inutility of the quinine in the present case ought not however to prejudice us too much against its use in similar cases. We have seen several examples in which it proved of decided efficacy in the practice of M. Chomel at the Hôtel Dieu. In all, the disease of the spleen was the result of intermittent fever. The quinine must be administered in large doses—10 or 12 grains daily—for a considerable length of time.—*Bulletin General.*

No mention is made of any of the preparations of iodine having been tried in the preceding cases.

We have found the hydriodate of potass, in combination with quinine, decidedly useful in several instances of enlarged spleen.

### On the Use of the Solid Nitrate of Silver in Gonorrhœal and other Discharges in Females.

It appears from the statements of M. Ratier, in a late number of the Lançette Français, that M. Ricord, the eminent surgeon of the Hôpital des Veneriens at Paris, was the first who employed the nitrate of silver, in the solid state, as an application in these diseases.

Since the announcement of this practice some years ago, in the pages of the Bulletin de Therapeutique, numerous medical men in other countries as well as in France, have confirmed the results of M. Ricord's experience. In Britain Drs. Balbirnie and Hannay have published memoirs on the subject, and recommended the practice in very high terms. The statements of the latter gentleman however seem to us to be too indiscriminate and unguarded ; for although the gonorrhœal and other discharges from the vagina in the female have been very remarkably benefited in our experience by the treatment alluded to, it is to be remembered that M. Ricord does not recommend its adoption, or at least does not promise much benefit from it, in all cases without exception. The differences in the character and severity of the complaint, in its duration, and in the idiosyncrasy and constitution of patients, forbid the scientific man from too general and too confident expectations in the cure of any disease.

We have another fault to object to Dr. Hannay; and this is that he has not adopted the use of the improved *porte-caustique,* recommended by M. Ricord, and trusts to the rude and clumsy expedient of a piece of the caustic inserted in a quill.

He indeed tells us that even if the caustic should slip out and remain in the vagina, we need have no apprehension of any inconvenience; but such an assurance is not likely to satisfy any cautious surgeon. The use of the *speculum vaginæ* is necessary to permit the caustic being safely and effectually conveyed to the os and cervix uteri. As the seat of the discharge may be either the neck of the womb, or merely the mucous membrane of the vagina, the mode of applying the caustic will vary to a certain extent in different examples. When the former is the case, if the interior orifice be sufficiently patent, the caustic should be introduced fairly within it, and the inner surface of the neck of the organ should be freely rubbed with it.

In withdrawing the instrument, the whole extent of the vaginal mucous surface also should be similarly treated.

When the uterine orifice is too narrow and confined to allow the introduction of the solid caustic, we should then use injections of the nitrate.

In such cases, where the morbid discharge proceeds from the vagina only, it is unnecessary to apply the caustic to the *os* or *cervix* of the uterus.

The treatment by the application of the solid nitrate of silver has been found of great benefit not only in old gonorrhœal and leucorrhœal discharges, but also in recent or acute gonorrhœa, before the inflammatory symptoms are subdued.

The nitrate, locally applied, is a most potent *antiphlogistic* remedy. We have witnessed numerous cases of acute gonorrhœa in the female cured by two or three applications of the solid nitrate to the mucous surface of the vagina: the burning uneasiness and pain induced usually cease after the first application. When the discharge has nearly ceased, M. Ricord recommends that a plug of dry charpie should be introduced and retained in the vagina; as he is of opinion that the mucous membrane recovers its healthy condition more quickly when its opposite surfaces are kept apart from each other; just in the same manner as the discharge in *balanitis* is so much diminished by putting a strip of dry lint around the glans penis.

However useful the treatment of Gonorrhœa, &c., by the application of the nitrate of silver has proved, M. Ricord is far from recommending its adoption, to the exclusion of other methods. The use of simple cooling injections, and of purgatives and low diet will suffice to cure many cases of the disease when recent; and the more chronic forms are often very effectually relieved by astringent washes, and by the exhibition of steel and other tonics internally. Perhaps, no medicine is, on the whole, more efficacious in improving the general health and in thus correcting the local disease in *old* leucorrhœal discharges than *steel*, in some form or another. Warm clothing too will often contribute to remove this troublesome class of complaints.

In reference to this point, M. Ricord says: 'how often have we seen most obstinate vaginal discharges quickly disappear, as soon as the use of warm stockings and flannel drawers had protected the feet and limbs from cold and moisture.'

In conclusion, we are informed that M. Ricord gives a decided preference to the application of the solid nitrate of silver to the mucous surface of the vagina (and also of the os tincæ in old obstinate cases) over the use of injections of this metallic salt. All that is necessary in most cases is simply to pass the caustic rapidly along the surface.

It has been observed that the slight irritation, thus induced, very often causes a premature coming on of the catamenia.—*Gazette des Hôpitaux.*

---

### M. Rayer on Diseases of the Kidneys.

This distinguished physician of La Charité has for some years past devoted much of his attention to the pathology of the Urino-poietic organs. In the course of last year he published the results of his enquiries in his "Traité des Maladies des Reins, etudiées en elles-memes, et dans leurs rapports avec les maladies des ureteres, de la vessie, de la prostate, et de l'urethre."

The high importance of examining the chemical condition of the urine in many cases, especially in all those whose nature and symptoms are obscure, has been recognized of late by all practical physicians. In the present day, no one ever omits to investigate the exact state of the thoracic organs, the heart as well as the respiratory organs, by means of auscultation; and the great value of the signs afforded by this mode of enquiry is too obvious to require any comments of ours at present.

The *seemingly astonishing increase* of diseases of the heart of late years is altogether attributable to the improved means of exploration now in use, and to the consequent greater accuracy of diagnosis.

It is unnecessary to say how much we owe to the labours of Laennec on this score.

No less important is the examination of the urine in the ætiological history of many diseases.

The important discovery of Dr. Bright that an albuminous state of this secretion is very frequently associated with, and symptomatic of, structural disease of the kidneys, has been one of the main causes of the attention paid, of recent years, to this subject. Some form of dropsical effusion, more especially anasarca, is a common accompaniment of this state of the urine; but it is not necessarily, or inevitably, so. The urine may be for a length of time charged with albumen, and yet scarcely any dropsical effusion may be present; and again, this effusion, although it has taken place, may be dispersed, and yet the urine may retain its diseased characters.

It is necessary, however, to observe that the urine does occasionally exhibit traces of the presence of albumen, when the kidneys are not at all affected. Such may be the case in certain inflammatory, or perhaps rather in the sequelæ of certain inflammatory, disorders, such as scarlatina, measles, &c.

But under such circumstances, the symptom does not continue long, the albumen is usually in small quantities, and there is no uneasiness or pain in either of the lumbar regions.

On the other hand, whenever the albuminous condition of the urine (*albuminuria*) continues for a length of time, and is accompanied with lumbar uneasiness, and with partial dropsy, there are strong grounds of suspicion that the kidneys are more or less affected with that disease, to which M. Rayer has given the name of *Nephritis Albuminosa.*

The following is the process recommended by M. Desir in his thesis, (de la presence de l'albumine, dans Dec. 1835), to detect the presence of albumen in the urine.

After filtering it, if it be troubled or thick, and adding a small portion of acid, if it be alkaline, it is to be exposed to heat, either in a tube over the flame of a lamp, or in an open vessel on a sand or water bath.

If nitric acid be employed, it should be added drop by drop.

By employing both methods, we shall succeed in detecting the smallest traces of albumen.

It is to be remembered that albuminous urine, if this be alkaline, will not coagulate by heat. If the albumen is in minute quantity, the urine becomes somewhat opaque: but the coagulation is instantaneous, whenever an acid is added. On the contrary, we occasionally meet with alkaline urine, which exhibits, on the application of heat, flocculi somewhat similar to those of albumen, but becomes clear again when an acid is added to it.

M. Rayer has investigated, with great minuteness, those morbid changes of the kidneys, in which the urine is found charged with albumen, and has represented them most faithfully and beautifully in the atlas of engravings accompanying his work.

He has come to the conclusion that all these changes are the products of chronic inflammatory action; and, upon this ground, he has designated them all under the general appellation of *Nephritis Albuminosa.*

M. Rayer has described, with great accuracy, the incipient changes in the structure of the kidneys. He says, that in the first or early stage of the disease, these organs are enlarged in size—their weight being increased from 4 or 5 oz.

(the average weight of a healthy kidney) to 8 or even 12 oz. Their consistence is firm without being hard; their colour is more or less deeply red, and often exhibits the appearance of being dotted with spots of a deeper hue than the rest of their surface. On cutting through a kidney in this state, we find that the increase in bulk arises from the tumefaction and congestion of the cortical substance.

This tissue exhibits a number of minute red points, similar to those observed on the surface, and corresponding for the most part, to the highly vascular glandules of Malpighi.

If to these considerations we add that, during life, the lumbar regions are always more or less tender on pressure, and that a regulated antiphlogistic regimen is of decided utility at this, the early, period of the disease, and that blood drawn usually exhibits a more or less decided buffy coat on its surface, we cannot surely hesitate to regard the early morbid change to be one that is strictly inflammatory.

The *second* stage of the disease is indicated by the following pathological condition. The volume and weight are still considerably increased; but the special character of this period is the admixture of pale and of congested spots (anemies et hyperemies), which gives a marbled aspect to the surface of the kidney. The tumefied cortical substance exhibits, on incision, a pale, yellowish, spotted appearance, while the inner or tubular substance is of a reddish brown colour.

In the *third* stage, the cortical substance presents a uniformly pale colour, sometimes of a yellowish rosy white, or of a hue still more pale, and similar to that of eel's flesh.

The partial vascular engorgement has given way to a more decided *anemia;* but the organ still remains tumefied, and exhibits the vestiges of an inflammation, where resolution has made no progress.

In the more advanced periods of this stage, we observe what has been called the granulated state of the kidneys.

The diseased organs are still more bulky and weighty than in health; their external surface is sprinkled over with minute dots of a milky-white or yellowish colour; but there is no prominence or unevenness to be felt by the finger. These small milky spots are designated by Dr. Bright *granulations.* Sometimes they are confined chiefly to the outer surface of the organ, while at other times they occupy the entire thickness of the cortical substance.

If the kidney be sliced across from its convex to its concave edge, the cortical substance—which is still swollen and more bulky than in health, especially in the prolongations between the cones—exhibits, as in the former stages of the disease, a generally *anæmic* and yellowish aspect, which contrasts very strikingly with the deep red colour of the tubular or inner portion.

Now these changes are all the results of inflammatory action. That such has existed is indicated by the increase of bulk and weight of the viscus, and by the gradual transition from a state of high sanguineous congestion to that of general anæmia and yellowish *degeneration.*

Although the kidneys are, in most cases, augmented in size in the disease, which we have been describing above, it is to be remembered that in certain instances they are actually smaller than in health.

When this is the case, their structure is usually firm and hard, and their surface uneven and irregular. There may perhaps be no white milky spots to be seen on the outside; but, on making an incision through the affected kidney, we generally perceive a certain number of them imbedded in the cortical substance.

From all these remarks it appears that in *Nephritis Albuminosa* it is the cortical substance of the kidneys, which is primarily and most seriously diseased. It is in it, that the vascular engorgement takes place, and that the granulations of Bright are formed. We may therefore say that the *Nephritis Albuminosa* is a *Nephritis* of the cortical substance.

With respect to the special characters of this form of Nephritis,—independently of its anatomical and pathological phenomena—they may be stated to be, 1st, the elimination and discharge from the system of albumen dissolved in the urine, and, 2ndly, the entire absence of any tendency to suppuration in the affected tissue—such as we observe in other forms of the disease; for example in the *Nephritis calculosa,* and the *Nephritis,* which not unfrequently is present in typhus and yellow-fever, &c. The discharge from the system of albumen by the urine must, in course of time, exert injurious consequences on the general health. The serum of the blood loses part of its specific gravity, as M. Rayer has discovered by examining the blood drawn from his patients. There is a marked tendency, in various parts of the body, to take on a chronic inflammatory action—for example the brain, pleuræ, and the abdominal viscera;—and the disposition to dropsical effusion is well known to every physician.

Our forefathers ascribed high value to the state of the urine as a means of *prognosis;* drawing, from its varying characters, conclusions as to the coction of the humours, the approach of crises or important changes, the influence of critical days, the gravity of the disorder, and the chances of recovery. In the present day, we study the urine as a means rather of *diagnosis,* than of *prognosis;* and, to arrive at accuracy of the former, we examine the secretion in its relations of acidity, alkalinity, and whether it contains pus, or albumen, or sugar, or spermatic animalculæ, &c.

In this way only can we hope to form accurate conclusions, regarding the true nature of a number of very obscure and puzzling cases.

In concluding this brief sketch of M. Rayer's researches, we cannot do better than highly recommend the perusal of the original work to the careful perusal of our practical readers.—*L'Experience.*

---

Illustrations of the Pathology of the Capsulæ Supra-renales. By M. Rayer.

M. Rayer, whose researches on the Diseases of the Kidneys we have just noticed, has recently published an account of his investigations of the morbid changes of these glands, the uses and functions of which have hitherto eluded the enquiries of all physiologists.

It appears that they are frequently the seat of an internal hæmorrhage, and of subsequent degeneration of their tissue.

We shall briefly report the particulars of some of the cases, which he has published.

Case 1. *Tumor in the Right Flank, produced by an enormous Apoplexy of the Capsula Infra-renalis.*

A woman, seventy-five years of age, was admitted in April 1836, into La Charité hospital, and died there in the course of the following month. Five years before, she had a severe attack of excruciating pain in the right renal region, extending thence towards the womb: micturition was frequently attended with difficulty and strangury. This attack lasted for nearly two months, and then subsided. Although she continued to experience occasionally a certain degree of uneasiness in the same spot, her health remained tolerably good, till within three months of her decease; when an attack, similar to the first one, supervened. The pain in the right loin was excruciating and extended downwards in the line of the thigh; micturition was painful and difficult; and the patient was much distressed with nausea and vomiting. Subsequently the affected limb became œdematous; and a tumor was discoverable in the right lumbar region: it was partly hard, and partly soft and fluctuating. The diagnosis was certainly obscure and difficult: the tumor, it was supposed, might be hydatidic or cancerous, or proceeding from dropsy of the ovarium.

On dissection, the whole extent of the right iliac fossa was occupied with an irregular-shaped tumor, situated between the right lobe of the liver and the edge of the os innominatum: it was nearly four pounds in weight: elastic and fluctuating on pressure, it discharged, when cut open, nearly two pounds of dark blood. The parietes of the sack were of a dark brown colour, and of a soft fleshy consistence. Upon a minute examination of its connexions, there could be no doubt that this large mass was formed by the supra-renal gland. What its genuine nature was, it was not easy to determine. We may style it an immense *apoplectic sac*, or perhaps rather *"une collection d'apoplexies multiples dans une meme poche."* The contained blood was partly liquid, and partly consisted of coagula, more or less organised. The right kidney was little, if at all, altered in any respect; and the left one, as well as its supra-renal gland, was perfectly healthy.

CASE 2. *Apoplexy of both Supra-renal Glands—Partial Disease of the Left Kidney and of the Bladder.*

A woman, 68 years of age, was admitted into La Charité in the beginning of the year 1836. She had been confined to bed for a fortnight with a troublesome bronchitic affection, which proved fatal. It would seem that no disease of the urinary viscera had been suspected.

*Dissection.* It is unnecessary to detail the pathological appearances found in the chest. On examining the kidneys, the cellular texture of both supra-renal glands was observed to be more or less infiltrated with dark blood; here and there, there was a distinct *"foyer apoplectique."* The structure of the kidneys themselves appeared to be quite healthy.

Bonetus, in his Sepulchretum, has detailed the particulars of a case, in which the left supra-renal gland had become an immense sac, filled with bloody semi-purulent matter; and Lieutaud alludes to two similar examples.

M. Rayer informs us that he has several times discovered a sanguinolent infiltration of the supra-renal tissue in new-born infants, who have died soon after birth. Twice he has met with the capsule transformed into a genuine sac or pouch, as large as a pigeon's egg, and full of blood.

Before dismissing the subject of *apoplexy of the supra-renal gland*, it may be useful to allude to the normal structure of the gland itself. Some anatomists have described it as hollow, or containing a cavity, and among those is Bichat, who uses these words:—"Chaque capsule surrenale n'est vraiement qu'une petite poche à parois parenchymateuses."

Others, on the other hand, such as Meckel, &c., assert that, in the normal state there is no cavity, and that, whenever such is found on dissection, it is the result "de la decomposition spontanée de la substance interne des capsules, qui a très-peu de consistence, ou de la destruction de cette meme substance par les manipulations, auxquelles on soumet l'organe en l'examinant."

To this opinion M. Rayer appears to incline, as he tell us that whenever he has met with any cavity in a supra-renal gland, it was always filled either with blood, or with a yellowish albuminous fluid, and that the parietes of the cavity presented such an appearance as to suggest to him that it was the result of an apoplectic effusion. He therefore thinks that the cavity, which we occasionally meet in the supra-renal gland, is not normal, but is the result of a rupture of its tissue—in most cases probably from hæmorrhage. The blood-vessels of this gland are exceedingly lax, and, therefore we may suppose, readily lacerable.

The other lesions, to which the supra-renal glands are subject, are few, as far as pathology instructs us—apoplexy or hæmorrhage being much more common than any other.

M. Rayer has seen one or two examples, in the fœtus, of a small abscess being formed in their structure. He has also met with tubercles in cases where the corresponding kidney was similarly diseased; with deposition of cancerous or cerebriform matter; and other observers have discovered a few cases of partial ossification, or conversion into a chalky substance. Meckel alludes to two instances, where he found the supra-renal glands much hypertrophied in individuals, who had been greatly addicted to venereal pleasures; and, of late years, the school of philosophical anatomists has directed our attention to the frequency of atrophy or imperfect development of their structure in acephalous infants. Müller, however, questions the accuracy of this statement; and, with Meckel, is rather inclined to believe that their development is in some manner connected with, and relative to, that of the generative organs.

(Such of our readers as wish to make themselves acquainted with the literary history of the supra-renal glands will do well to consult M. Rayer's memoir, which they will find in last October number of *l'Experience, ou Journal de Medecine et de Chirurgie.*

---

### Cases of Glanders in the Human Subject.

Corporal Rudorff, 25 years of age, when admitted on the 10th of July into the military hospital, had for about five weeks previously been suffering from intermittent fever—his health, even before that period, having not been good for a length of time.

For some days before his admission, he had experienced sharp pains in the joints of the lower limbs, accompanied with a feeling of weight and heaviness in those parts, and with severe headache. When he came into the hospital these symptoms continued, the face was flushed, the skin was hot and dry, pulse hard and rapid, and there was also great thirst.

The disorder was regarded as acute rheumatism, or as inflammatory fever. The patient was ordered to be bled, purged, and kept on a cooling diet.

The venesection procured great relief; the blood being very buffy.

On the 12th, numerous furuncular tumors appeared on both legs; they did not present any outward marks of inflammation, their surface being of a pale blue colour: they were very tender on pressure, and were attended with a burning stinging pain. They felt as if filled with a fluid matter, and as if this extended deep among the muscles.

The pyrexia and rheumatismal pains returned; and further depletion was therefore necessary. The fever began to assume more and more of a nervous type, being attended with much delirium and stupor, and the tongue covered with a brown crust. Several new tumors made their appearance on the thighs, arms, and neck. Some of these were hard and exquisitely tender, while others were less sensitive, and felt soft and fluctuating. The symptoms became progressively more and more unfavourable, the patient lying constantly in a state either of delirium or of coma, and exhibiting all the worst features of typhus gravior. He died on the 18th—being the eighth day after his admission.

*Dissection.* On opening many of the subcutaneous tumors, they were found to contain, some a thick yellow pus; others a dark-coloured fluid like chocolate. This matter was also infiltrated into the substance of the adjacent muscles, and at some points reached even to the subjacent bone. The blood in the veins was remarkably watery, *decoloré*, and in the right auricle seemed as if it was mixed with a viscid fluid. The right crural vein was coated here and there, on its internal surface, with a puriform exsudation.

*Remarks.* The preceding case exhibited all the symptoms of *glanders*, such as it has been observed in the human subject. The patient had had the charge of several glandered horses, before the commencement of his illness.

*Case* 2. A pupil of the Military Veterinary School had been attending some glandered horses, and had frequently handled them, when there was a small wound on one of his fingers. He began to experience a pain in this hand, and to become generally feverish. The pain speedily extended to other parts of the body, more especially the joints, and the affection resembled altogether an attack of rheumatism. When admitted into the hospital on the 3rd of February, several of the joints both of the upper and lower extremities exhibited a slight tumefaction, and the system was labouring under general pyrexia. He was bled, purged, &c. with temporary relief to the symptoms.

On the 12th, several circumscribed puffy tumors were felt under the skin of the arms, legs, and head, although no outward redness was visible.

He was again bled and put on a course of calomel and opium.

A diarrhœa, accompanied with extreme prostration, supervened; and symptoms of cerebral oppression followed: in short, all the usual characters of aggravated typhus were present.

Some of the furuncular tumors were exquisitely painful, especially one as large as a nut over the left parietal bone. The face was covered with numerous miliary vesicles.

The patient died on the 4th of March.

*Dissection.* All the encephaloid vessels were gorged with blood, and there was a sanguinolent effusion in the cavities of the brain and spinal-marrow. A similar effusion was present in the pleural and pericardial cavities.

Numerous tumors, filled with a dark sanious fluid, were discovered in the substance of the muscles of the upper extremities, and also in one or two muscles of the legs: the surrounding muscular tissue being more or less softened and disorganized.—*Medicinishe Zeitung, Mai* 1837.

---

### ON THE CONTAGION OF THE VARIOLA, DURING 1836, IN HAISCHBERG IN GERMANY.

Dr. Schaeffer has communicated a long article on the above subject, closing it with the following conclusions.

1. Vaccination, as a preservative against the small-pox, has appeared to be in a direct relation with the number of *scars* visible on the arms of the individuals. Thus in 43 vaccinated persons, who have caught the small-pox, there were, in all, only 130 scars; whereas in other 38 persons, who have escaped, although they had been repeatedly exposed to infection, there were 211 scars counted.

2. Vaccination is far from having the same preservative influence on all persons. In some a single cicatrix seems to prove a sufficient security against the small-pox; while others, whose arms have exhibited half-a-dozen of genuine scars, have been seized with the disease.

3. It is very difficult, if not quite impossible, to pronounce any characteristic signs of true genuine cicatrices.

Of 43 vaccinated persons, who have taken the small-pox, 14 exhibited cicatrices perfectly *normal*; in 25 they were more or less *abnormal*, or irregular; in 4 none at all could be discovered.

On the other hand out of 38 vaccinated persons, who had resisted the infection, 34 presented normal cicatrices; in 3 they were more or less indistinct, and in the remaining one no traces were visible.

4. The majority of vaccinated persons, who have been attacked with small-pox, have been from 20 to 30 years of age. The severity of the disease has been almost always in a direct ratio with the length of the interval elapsed since vaccination had been performed. In general the disease was more severe in the more aged than in the youthful. In one case the small-pox declared itself in an adult immediately after a normal vaccination; and in another case it showed itself, in a slight degree, on the eighth day after vaccination—the progress of which however was not interrupted.

5. A great number of persons have been submitted to re-vaccination; and of these not one has been attacked with small-pox. Moreover all those also, in whom re-vaccination has not produced any effect, have escaped, although many of them have been much exposed to the contagion—a strong argument in favour of the opinion, that when re-vaccination does not *take*, the liability to catch the small-pox is extinguished.—*Rust's Magazin fur die ges: Heilkunde.*

---

### THERAPEUTIC INFLUENCE OF COMPRESSION OF THE LARGE BLOOD-VESSELS IN NEURALGIA, &c.

M. Dezeimeris, the author of the memoir from which the following observations are drawn, appears to be much more deeply read in British medical literature than any of his countrymen, with the exception of two or three, such as MM. Rayer, Velpeau, &c.

He points to the late Dr. Parry of Bath, as being the first who ascertained and announced the curative effects of compression of the large arteries; and more especially of compression of the carotid arteries in various cephalic diseases, as severe headache, epilepsy, convulsions, &c.

M. Dezeimeris's observations apply chiefly to the efficacy of such compression in neuralgic affections of the face and head. The following two cases appear to us to be very interesting.

*Case* 1.—Madame C., 34 years of age, and of a rather feeble constitution, determined, after the loss of her parents in 1814, to retire to a convent. There she spent five years in fasting and various penances; the effect of which was to impair her health very greatly.

It is now about six years ago since she experienced the first attack of neuralgia, which came on after deep and protracted chagrin. The paroxysm of pain was quotidian, returning almost regularly between three and five o'clock in the morning. The use of the sulphate of quinine effectually cured it.

Three years subsequently, and again after severe mental distress, Madame C. was seized with gastralgia, accompanied with bulimia. These symptoms yielded to the use of pills composed of opium, magnesia and subnitrate of bismuth.

In the Spring of 1836 she had a return of facial neuralgia, which was not confined to one spot, being sometimes supra-orbital, and at other times infra-orbital or maxillary, or seated in the ear. It very rarely exhibited any regularly intermittent type; and hence resisted the quinine. Various narcotic remedies, employed endermically as well as internally, were tried, but with only temporary and partial success; and the disease did not fairly cease, untill a suppurative eruption made its appearance on the ear.

The last attack of the disease was in the Autumn of the same year. This time it occupied almost the whole right side of the face, and although not so severe as on the former occasions, it resisted every means that were employed.

The physician in attendance having accidentally spoken to M. Dezeimeris respecting this case, he, (the latter) suggested to him to try the effects of compression of the carotids. At this time the pains were so excruciating as to force the patient to scream out, and the integuments of the affected part were red, swollen, and shining. Upon firm pressure being made over the right carotid artery for two or three minutes, the facial congestion rapidly subsided, the pain became almost entirely assuaged, and the patient felt a soothing drowsiness creep over her.

When the compression was removed, the pain returned, but in a less aggravated degree; and again it was checked by re-applying the finger. For the following three or four days the sufferings of the patient were much less than they had been for a length of time, recurring at intervals only and with diminished severity. At length an eruption broke out in the right side of the face; and since then there has been no renewal of the pain.

*Case 2nd* was communicated to the author by M. Rayer.

A young female, of a highly nervous temperament, had been subject, for several years, to attacks of excruciating neuralgic pain in the right side of the face.

During the paroxysms, the suffering was so intense that she was scarcely conscious what she was about. All classes of medicines had been tried, but without any decided benefit.

M. Rayer, at the suggestion of M. Dezeimeris, tried compression of the corresponding carotid artery. After being continued for about twenty minutes, the pain was very greatly relieved. On the threatened approach of the next attack—for the paroxysm was usually preceded by a feeling of stiffness and numbness in the left temple—compression with the fingers over the left carotid, until the circulation through the corresponding temporal artery was sensibly diminished, was at once resorted to; and with the effect of preventing the accession of the fit. Since that period the return of the fits has not indeed been delayed or prevented; but, adds M. Rayer, the pain, which was formerly excruciating, has become so supportable that I may say that the paroxysm *miscarries* each time.

M. Dezeimeris then alludes to the curative effects of compression of the carotid arteries in some cases of convulsions and epilepsy; and narrates, without however approving of the practice, the three cases in which Mr. Preston tied the common carotid artery to relieve certain cephalic affections. —(*Trans. of Med. and Phys. Society of Calcutta.*)

He mentions also some of the observations of Dr. Cooke (History and Treatment of Epilepsy, London, 1823), to prove the benefit of compression of the carotids in many cases of this disease. We shall close these remarks with the short notice of a case of *Coma Vigil*, in which M. Rayer employed compression with advantage.

This case occurred in a young woman, who for many years had suffered from various forms of hysterical disease. Latterly she had become affected with a species of coma, which returned every day, and which usually lasted for several hours.

If she was aroused from this state, she became vehemently excited and convulsed. At the suggestion of M. Dezeimeris, M. Rayer tried the effect of compressing the carotids during the attack of coma. It was not continued for more than two or three minutes, before all symptoms of stupor ceased. The same practice was adopted several times afterwards, and always with the same effects.—*L'Experience.*

### UTILITY OF ERGOT OF RYE IN PARALYTIC AFFECTIONS.

It is reasonable to presume that the *modus operandi* of this remedy in tardy parturition, Amenorrhœa, &c., is by its stimulant influence on the lower extremity of the spinal-marrow. Hence some physicians have been led to try the use of it in some case of *paraplegia;* and, according to their reports, decided benefit has been thus obtained. It seems to have no influence in *hemiplegia:* the seat of that more unfavourable species of palsy being either in the brain itself, or in the cervical portion of the medulla.

The Ergot has been employed with marked benefit in that form of *paraplegia*, to which infants during dentition and young children are subject.

Adults too have derived equal benefit from its use. Thus M. Ducros of Marseilles mentions the case of a sailor who, by falling from the rigging of his ship, lost the use of his lower limbs completely. Moxas, and other means, had been unsuccessfully tried by Professor Delpech; and nothing seemed to do any good but the Ergot of Rye, under the administration of which he quite recovered.

The usual dose, which it is proper to commence with, is six or seven grains: this should be raised gradually to thirty or forty grains, or until the patient begins to feel prickings, and a sense of formication in the limbs, somewhat similar to what are produced by the use of the Nux Vomica.

An excellent adjuvant of its remedial virtues is the occasional exhibition of a turpentine enema.

Whenever we administer the Ergot of Rye for a length of time, it is necessary to guard against the *septic* influence of the agent on the system, by recommending the use of a generous animal diet, &c. &c. M. Ducros mentions a case in which sloughing of the heel supervened: by proper means, however, both the gangrene and the paralysis were cured.—*La Lançette Française.*

### RESEARCHES ON THE CAUSES OF ABORTION, BY MADAME BOIVIN.

Madame Boivin—the distinguished *sage-femme* of Paris, Doctor of Medicine, and chief inspectress of the Royal Maison de Santé, &c. has recently published some excellent remarks on "One of the most frequent and least known causes of Abortion." Her researches have been evidently conducted with great skill and fairness, alike in the examination of the symptoms during life, and of the morbid appearances found on dissection. We cannot do better than report two or three of her illustrative cases, for the purpose of pointing out the nature of the *cause*, which in her opinion gives rise so frequently to miscarriages.

" Madame Kall, twenty-seven years of age, mother of three children, was seized with pleuritic symptoms after exposure to cold. Miscarriage came on: the fœtus was about five months old. She died on the 10th day after the commencement of her illness.

*Dissection.* (We shall confine our remarks to the state of the uterus and its appendages). The broad ligaments, the Eustachian tubes, and the ovaries, were grouped or matted together, and adhered to the posterior surface of the uterus. The adhesion was so close, as to require the use of the scalpel to sever it. In the conglomerated mass numerous small tubercles, varying in size from a millet-seed to a pea, were discovered.

It is evident that, *such being the condition of the uterine appendages, the womb could not expand and develop itself, but with extreme difficulty.* Abortion must therefore have come on, although no affection of the chest had supervened. The uterine ligaments being morbidly affected could not readily expand and yield to the gradually-increasing distention of the womb: their resistance became the cause of excitation, and a miscarriage was the consequence."

From these short details our readers will be able to understand the reasonings of Madame Boivin, as respect *one of the most frequent and least known causes of abortion.* She adduces many other examples to prove the existence of some morbid change of the uterine appendages in cases of miscarriage. This change, whatever may be its character or appearance on dissection, seems to be almost always the result of some slow unhealthy inflammation. In some cases, the agglutination of the uterine appendages is found associated with the formation of purulent deposits between the uterus and rectum. Such cases usually occur in scrofulous subjects.

It may now be asked, is there any method of ascertaining, or even of suspecting, the existence of a morbid change in the uterine appendages during life? and, if so, are there any remedial means to remove it?

We must confess that the diagnosis of such a disease is always most difficult and uncertain. The following symptoms may, however, be mentioned as, to a certain degree, indicative of its presence. Menstruation is usually attended with severe pain and suffering; there is often a continual bearing-down and sense of dragging as well during menstruation as during micturition; the evacuation of the bowels too occasions the same feeling; there is often an acute or a dull oppressive pain in one or both groins, extending upwards to the loins and downwards to the limbs; and there is always more or less of leucorrhœal discharge.*

With respect to the treatment of such cases, Madame Boivin informs us that the local use of mercury—the inunc-

* There is one very important symptom to be ascertained by manual examination, which especially merits the attention of the physician—we allude to the frequent partial displacement of the neck and orifice of the uterus, and to the *diminution of its natural mobility*, when pressure is made on it by the finger.

The following remarks by Madame Boivin appear to us highly interesting: " As in the morbid affection of the uterine appendages it is rare that the womb retains its normal degree of mobility, it is therefore of great importance in all cases of irregular menstruation, or of obstinate leucorrhœa, to determine if the womb, independently of the healthy condition of its orifice, preserves all the freedom of movement which results from the normal state of its ligaments, and of the ovaries and Eustachian tubes."

tion of the mercurial ointment on the groins, thighs, &c.—continued so long as gently to affect the system, will often succeed, not only in restoring the health of the patient, but also in removing the tendency to subsequent miscarriages.

Madame Boivin does justice to Dr. Granville of London, in giving him the credit of having suggested this practice in his "Obstetrical Report of the Westminster Dispensary," published many years ago. Dr. G. adduced several cases, which very satisfactorily proved that the tendency to miscarriages may very often be checked by the *mercurial treatment*, in the way of inunction.

The other remedy, recommended by Madame Boivin, is the hydriodate of potash.—*Recherches sur l'Avortement, par Madame Boivin.*

---

### Irregularities of the Bowels, a frequent Cause of Uterine Affections. Pernicious Effect of a Boarding-school Life.

Many, if not most, of the troublesome affections of the uterus and of the rest of the generative organs are attributable *primarily* to derangement of the gastro-intestinal functions. This is especially apt to be the case in young females of a lax, lymphatic and scrofulous constitution: their appetite is usually irregular, and often bizarre, and their bowels are almost always sluggish and constipated. The system of education pursued in the present day unfortunately fosters, if it does not actually occasion, this state of things.

Little or no attention is paid in any boarding-school to the *physical education* of the girls: their occupations are almost all sedentary; their minds are much more exercised than their bodies; and even the very amusements, which are allowed them, are subjected to as strict a discipline as their regular studies. They become poor, feeble, pale creatures; their catamenial discharge is never quite healthy nor regular; and, when they become married, they either become the victims of frequent miscarriages, or else their offspring is puny and miserable.

When we consider that all the girls of a boarding-house are, without regard to difference of age or constitution, subjected to the same discipline, made to eat the same food, and altogether drilled in the same manner, how can we be surprised that so many of them, on leaving the establishment, return home with the seeds of much suffering and ill-health, deeply laid and ready to burst out when Nature designs them for the more important functions of their sex.

Of all the evils of a boarding-school life, perhaps none is so essentially pernicious as the neglect—arising from a variety of causes—of a regular action of the bowels. Almost every girl in such establishments suffers more or less from constipation. As they approach the age of puberty, this disposition becomes more confirmed; the abdomen becomes fuller; the large intestines especially acquire greater size; the pelvis is now more capacious—all these physical changes induce a greater tendency to extreme inaction of the bowels, and to the consequent accumulation of large quantities of fæcal matter in their cavities.

Now no portion of the intestines is so apt to suffer from this influence as the sigmoid flexure of the colon and the rectum. Independently of the injurious effect of alvine accumulations on the general health, the distention of the lower gut must necessarily cause a compression and a consequent congestion of the hæmorrhoidal vessels; the blood is thus obstructed in its free return. Hence the circulation not only in the rectum, but also in the uterus and its appendages, becomes gradually more or less deranged; thus laying the foundation for many of the most obscure and unmanageable affections of the uterine system—such as obstinate leucorrhœa, tendency to miscarriage, &c. &c. Many cases of nymphomania also are attributable to this same cause. The irritation extends from the rectum to the vagina and vulva; and hence, unless proper means are employed to remove the primary evil, all local palliatives to the seat of the distress are delusive and unsatisfactory.

If such be the state of health before marriage, many circumstances, mental as well as corporeal, occur afterwards to aggravate, at least very frequently, the morbid disposition.

What with fatigue of body, disquietude of mind, the excitement from sexual intercourse, &c.—all these causes tend to induce either a feverish irritability, or a languid congestion of the whole system.

As a matter of course, the organs which had been weak and ailing before, suffer most; and thus we may account for the numerous diseases of the uterine system which we meet with in the pre-present day, more especially in large towns.

Mad. Boivin remarks:—"The state, into which the whole organization has fallen in such cases as we have been alluding to, gives rise to irregular and diseased secretions, and to various morbid growths and degenerations of tissue, especially in the mammæ, and in the uterus and its appendages."*

Having thus explained her views respecting the morbid tendencies of the uterine system, our authoress goes on to observe:—

"I do not agree with those who believe that pregnancy, miscarriages, and difficult parturition are the most frequent cause of the diseases of the uterus. I am rather inclined to adopt the reverse of the proposition, and to assert that *certain morbid states*—the etiology of which we have explained above—of *the uterus and its appendages, are the most frequent causes of premature and abortive labours.*"—*Researches sur l'Avortement, par Madame Boivin.*

---

* We have had occasion, says Mad. B., to see fine young women, who exhibited all the characters of maidenhood, extremely subject to catamenial irregularities, and each labouring under a scirrhous affection of the mammæ. Three of these patients were operated upon by the Baron Dubois: their respective ages were 22, 25, and 38.

### Notice of the Hôtel Dieu of Paris.

The Hôtel Dieu of Paris is probably the oldest hospital in Europe. It was founded about the year 660, for the refuge and assistance of pilgrims, and the sick and destitute of the metropolis. Its motto was, *medicus et hospes.* At certain seasons it was so crowded that we are told that, at the beginning of last century, there was sometimes upwards of 9000 inmates, of all ages, sexes, and conditions in it, at one time. There were wards for lying-in women, children, venereal patients, and the insane. But, after the revolution, a great improvement in all these arrangements took place. The lunatics were transferred to the Salpetriere and Charenton Hospitals, and subsequently also to the Bicetre; the Maison d'Accouchemens and the Maternité received the obstetrical cases, while the children were established in a separate hospital, Hôpital des Enfans Malades; a venereal hospital was set apart for syphilitic cases; the other hospitals of the metropolis, viz. Necker, Cochin, Beaujon, La Charité, La Pitié, St. Antoine, St. Louis, &c. were greatly enlarged and improved; and, lastly, a Central Board (Bureau Central) was instituted to superintend the distribution of the various patients.

By these arrangements the crowded state of the Hôtel Dieu was reduced to 1800, 1200, and lastly, to 1000—which last number may be stated to be the average amount of patients in its wards at the present time.

The administrative service of this great hospital consists of a director, a treasurer, and four secretaries.

The medical staff, or "service de santé," consists of ten physicians, three surgeons, one chief clerk, *chef de clinique,* nine physicians' in-door clerks, *eleves internes en medecine,* ten surgeons' in-door clerks, 124 pupils, one apothecary and an assistant, and ten in-door pupils of pharmacy.

The sisters of charity, who act as nurses here, belong to the order of St. Augustin: of these, there are 32 matrons (*meres*), and 12 novices.

The physicians visit their patients every morning at six o'clock, assisted by their "*internes*," and their "externes;" and the resident physician visits all the wards each evening. All cases of urgency and danger are attended to by two of the *internes*, who keep watch night and day.

Of all the hospitals in Paris, the Hôtel Dieu is that which is best supplied with water. The author of the paper, from which we draw our remarks, candidly admits however that Paris, when compared with London as to the supply of this necessary of life, is still "*dans l'enfance de l'art.*" It is generally supposed that London consumes upwards of 100 times as much water as Paris. The chief supply to the Hôtel Dieu is from the pump of Notre Dame. But it is not possible for us to follow the author through all his minute details of the general economy of this vast establishment. We must therefore refer our readers for further particulars to the number for last November of the—*Annales d'Hygiene.*

---

### WOUND OF THE EAR FROM THE INSERTION OF A KNITTING NEEDLE —DEATH.

A youth, in mere sport, had introduced the end of a knitting-needle into the left ear of one of his companions, who, by suddenly moving his head, most unfortunately had caused the instrument to pass deeply in, and to wound the inner apparatus. He uttered a piercing cry, and fell down quite insensible. He was bled and freely purged; his consciousness gradually returned. On the following day however he was delirious, and on the third he became comatose. The nature and extent of the local injury, could not as a matter of course, be ascertained. Convulsive twitches of the face and body supervened; but the patient continued altogether insensible. He died on the evening of the fourth day.

*Dissection.* The outer vestibule, or cavity of the concha, exhibited no lesion, and the external auditory canal also was perfectly sound. The *membrana tympani* was so much lacerated, that only a few shreds remained attached to the bony ring. The *tympanum* itself was filled with pus; the *malleus* and *incus* lay loose and separated from their connexions, the ligaments and muscles being destroyed: no trace of the *stapes* could be discovered. The *fenestra ovalis*, situated immediately opposite to the *membrana tympani*, was found open, and the small membrane or ligament, which unites the base of the *stapes* to it, had disappeared. The nerve, which traverses the tympanitic cavity, was divided across. The inner ear being now carefully examined, the soft parts contained in the vestibule—the three *ampullæ*, and the three soft portions of the acoustic nerve—were found to be blended confusedly together. Among these parts, a fragment of the *stapes* was discovered. The *cochlea* and three semi-circular canals exhibited nothing remarkable, except high injection of their capillary vessels.

On opening the cranium, the dura mater was found to be thickened and highly vascular; the arachnoid exhibited traces of intense inflammation; some sero-purulent matter lay on the surface of the pia mater, and had partially insinuated itself between the convolutions of the brain. The cerebral substance also appeared to have suffered from inflammatory action. A small effusion of blood was discovered on the upper surface of the *petrous bone.*—*La Lançette Française.*

---

### BLISTERS IN UNFAVOURABLE CASES OF ERYSIPELAS OF THE SCALP.

The following case is worthy of notice. A healthy robust man, 31 years of age, was carried to La Charité, in consequence of an accident which he had met with by a quantity of earth and stones rolling down upon him, when engaged in digging a deep pit at the side of the Bicetre. No limb was broken; but he had received severe contusions in various parts of his body. The scalp was lacerated at several points, and the left ear had been quite detached from its connexions. The poor fellow was labouring under symptoms, which indicated cerebral compression. He was therefore freely bled, and leeched; but without any decided benefit. The scalp had become affected with erysipelatous inflammation, which had extended down over the face and neck: delirium came on; and on the 5th or 6th day he was *à toute extremité*. The pulse was almost imperceptible, and the tongue was dry and covered with a black crust. It was generally expected that he would die in the course of the day.

In this state of things, M. Velpeau ordered that the whole scalp should be covered with a blister. On the following day, what was the surprize of the pupils at finding the patient perfectly sensible and composed! The pulse had risen in strength and had become regular; and all the other symptoms had improved. *En verité n'est-ce pas là un miracle?*

Four or five abscesses formed in the substance of the scalp and also in the two eyelids; these were opened and subsequently cicatrised. Ultimately the man quite recovered.—*Bulletin General.*

---

### On the Treatment of Fractures, by M. Velpeau.

The object of the indefatigable surgeon of La Charité is to shew that a patient, after the broken limb has been secured in an "apparatus inamovible," may be permitted to walk about on crutches, with perfect safety.

He premises his remarks by observing that a very popular error still exists, that it is of great consequence that a fractured limb should be *set*, as soon after the occurrence of the accident as possible; and hence it is generally supposed, by unprofessional persons, that the sooner that the displaced ends of the bone are rectified, the more favourable will be the progress of the case. "Unless," says M. Velpeau, "some serious complication exists, it is almost quite indifferent whether the apparatus be applied at the end of a few minutes or of the first twenty-four hours. Up to this time there is nothing to be dreaded from delay. I wish to insist much on this point, in order to prevent the unnecessary alarm in a family, which is excited on all occasions of a broken bone."

Some surgeons, however, carry this system of delay much too far, when they recommend that a fractured limb be left without any apparatus for the first eight or ten days. This period, they pretend to say, is necessary to combat the swelling and inflammatory action of the surrounding soft parts; and, as the agglutinative and consolidative process of incipient ossification does not commence till the second week or so, it is useless, they add, to keep the ends of the bone in exact apposition.

M. Velpeau very properly condemns this line of practice. How can the sharp ends of the bone, he asks, remain in contact with the soft parts without irritating them, and exciting spasm and inflammation? and as for the objection that the compression from the immediate application of splints and bandages is apt to induce gangrenous inflammation, it holds good only of the injudicious, and not of the scientific, employment of the means.

Indeed, ever since the commencement of my practice, I have uniformly acted on the principle of immediately reducing the displaced bones, and of then surrounding the limb with discutient compresses, and with a moderately-compressive bandage, carefully applied from the fingers or toes upwards along the whole extent of the injured limb. These objects being effected, the only other indication was, in my opinion, to maintain the limb in an immoveable, and as easy a position as possible. By following these principles of practice, I (M. Velpeau) have succeeded in curing some most severe and unfavourable cases of fractured limbs, even when the bone had projected through the integuments, and great infiltration had taken place into the soft parts.

M. Velpeau gave a very fair trial to the practice recommended by Baron

Larrey—of wetting the bandages, when first applied, with an agglutinative fluid composed of liq. plumbi, camphorated spirit, and the white of eggs—and was inclined for some time to think very favourably of it. But of late years he has discontinued it, in consequence of the following objections :—

1. When the tumefaction of the limb subsides, a hollow is left between it and the bandage.

2. It is difficult to remove the bandage, in case such a removal be expedient.

The latter of these objections is, in a great measure, obviated by the proposal of M. Seutin, a distinguished surgeon of Brussels, to employ a mere solution of isinglass or of starch in place of the liquid of Baron Larrey; the former being as firmly agglutinative, and much more readily softened and removeable than the latter.

M. Velpeau approves of the suggestion ; and the following is the mode in which he has availed himself of it in the apparatus which he now generally uses in the treatment of fractures.

Having reduced the displaced bone by appropriate extension, he applies a roller from the toes up to the knee : this is to be well wetted with a strong solution of starch ; and then it is to be re-applied backwards from the knee down to the toes. The opposite surfaces of the roller become very quickly glued to each other, so that it is not at all necessary to use pins to keep any of the turns together.

It is a good plan to place some wadding along each side of the tendo-achillis, so as to fill up the hollow between the two layers of the bandage. He then applies two or three pasteboard splints, moistened in the starch solution, on each side of the limb, and one to the sole of the foot. These are maintained in strict apposition to the limb by means of another roller, which is also wetted with the liquid, so as to cause it to adhere firmly to the splint. When this has dried, in the course of two or three days, the limb is found to be cased in a solid greave, so to speak, of pasteboard and bandage, and all risk of the displacement of the fractured ends of the bone is effectually prevented. Hence the patient may not only move or turn the limb in bed with perfect safety, but he may even rise and walk about on crutches, whenever the desiccation of the apparatus is complete—and this is usually the case by the third or fourth day.

M. Velpeau assures us that he has treated upwards of sixty cases of fracture, in the manner now described, with most complete success, and with far less trouble, both to surgeon and patient, than with the ordinary apparatus. He alludes also to the great success of M. Seutin in Brussels, who has now for some years trusted entirely, in his extensive surgical practice, to the "methode par compression inamovible," in all cases of fracture of the upper and lower extremities.—*L'Experience, Journal de Medecine et de Chirurgie.*

---

### M. GERDY ON BANDAGES AND OTHER SURGICAL APPARATUS. (Review.)

M. Gerdy, one of the surgeons of the Hôpital St. Louis and the Professor of Surgical Pathology to the Faculty of Medicine at Paris, published in 1826 his *Traité des Bandages et Appareils de Pensement,* with an Atlas of Engravings. The Second Edition, which is quite recently published, contains many additions and improvements.

The first part of the work is occupied with a description of the various sorts of *bandages,* properly so called, and the second makes us acquainted with the numerous and diversified machines and mechanical contrivances for fractures, curvatures, &c. &c.

The present edition will be found to contain a description of all the valuable new apparatuses, which have been recommended within the last ten years.

Thus, he particularly describes the method of treating fractures by M. Mayor of Geneva ; the immovable apparatus of Baron Larrey, of M. Seutin and others, for the same purpose ; the various kinds of trusses and herniary bandages; certain novel contrivances for spinal deformities, &c. &c.

The historical remarks appended to each chapter will enable the reader to apply, if he wishes, to the original sources of information ; and the author has particularly dwelt on many of the surgical apparatuses of the ancients, more especially of Galen and Oribazes.

The descriptions of the different articles, instruments, &c. are perspicuous, without being too minute ; and they are always accompanied with practical reflections on the advantages and inconveniences of each.

The Atlas contains seventeen well designed plates—neatly and elegantly engraved.

---

M. Boucheron on the Hair.
(Review.)

The work of our author is entitled *Traité Anatomique, Physiologique, et Pathologique du Systeme Pileux, et en particulier des Cheveux et de la Barbe*, p. 158, Paris, 1837. There is a good deal of amusing information in its pages; and as the subject of the hair is not generally much attended to in physiological works, the curious reader will find it well worth his while to peruse M. Boucheron's brochure.

A considerable space is devoted to the interesting topic of the reproduction of the hair in cases of *baldness*.

From minute enquiries the author has come to the conclusion that, in general, the bulbs of the hair are only partly atrophied in the tissue of the true skin or dermis,—a circumstance which does not prevent them from recovering their original functions, and from re-secreting the horny matter which forms the hair, under the influence of certain peculiar stimuli.

"This proposition," he says, "will cease to appear at all strange, if we call to mind the remarkable circumstance of the occasional formation of accidental hairs in various parts of the body.

Every one has no doubt heard, for example, of masses of hair being sometimes found in cysts, and on cicatrices and erectile tumors.

If repeated experiments had not incontestably proved that after the forcible extraction of the bulbs (as, for example, in the treatment of some cases of *tinea*) the hairs may regrow by other bulbs—whether these bulbs are new formations, or whether they pre-existed in the state of germs—the mere observation of accidental hairs should be sufficient to argue the possibility of such an occurrence. As pointing to the same conclusion, we may allude to the circumstance, that the almost imperceptible down, which we observe on the face of some women, usually assumes such an increase, after the age of 40 or 45 years, that they become *etonnemment barbues*.

Why therefore should it be impossible to re-animate by appropriate means the hair in cases of baldness, and to excite it to increased activity of growth on the beardless skin of certain faces?"

From the circumstance of the bulbs of the hair being obliquely and confusedly implanted in the tissue of the *dermis*, M. Boucheron explains why, *when one straggling white hair is pulled out from the head, for example, the neighbouring hairs so speedily blanche in their turn;* "for," says he, "the extraction of one bulb disturbs and injures the adjacent bulbs, enfeebling their vitality, and thus disposing them to weakness and decay."

There are hairs which, being feeble originally, do not pass through the epidermis ; they roll or curve themselves under it, having not sufficient strength to penetrate this envelope.

If however, from any cause, the vital energy of this portion of the skin is augmented, it may become gradually more and more hairy, from having been quite bald and smooth.

M. Boucheron alludes to a curious case, which occurred in the practice of M. Rayer, and in which the inner surface of the thigh, which had hitherto been nearly quite smooth, became covered with numerous stiff hairs, after an attack of phlegmonous inflammation. The same phenomenon is occasionally witnessed as a consequence of blistering.

M. Boucheron has minutely investigated the subject of the colour of the hair. He attributes it to a peculiar animal oil, secreted by the bulbs, and varying consequently in its properties in different individuals. It is to a change in the colour of this oily matter—arising from a variety of causes which tend to enfeeble the general power of the system—such as advance of years, intense study, grief, &c.—that M. Boucheron attributes the whitening of the hair.

The author gives a minute and very interesting description of the diseases of the *systeme pileux.* We must refer the curious reader to the original work for details—they will well repay the trouble of perusal.

---

### Dr. Caspar on the Duration of Human Life, &c. &c. (Review).

Dr. Caspar of Berlin published a long and valuable memoir on the above subject about two years ago; and as he is now considered a standard authority on this branch of statistical knowledge, we shall borrow a few extracts from his *Wahrscheinliche Lebensdauer.*

After having examined with much minuteness the opinions of different authors as to the average duration of human life, and as to the most satisfactory method of ascertaining such a result, he announces his own doctrine in the following proposition: *The proportion of births to the population in any place expresses almost exactly the medium or average duration of life there.*

For example, suppose that this proportion is in the ratio of 1 to 28—then the average life of the inhabitants of the place will be found to be 28 years.

If this rule be correct, it must follow that the duration of life increases and diminishes in a population, according as their fecundity is greater or less; so that man—if not as an individual, at least as a member of the mass—may be said to have it in his own power to lengthen or to abridge his life.

This, if true, is indeed a proportion of great importance in political economy.

To prove that the mortality is in a direct ratio with the fecundity of any population, and consequently that governments—seeing that the force of states consists not so much on the mere number, as on the strength, fecundity, and longevity, of their inhabitants—ought not to favour or encourage an over-abundant population, the author has collected together a vast number of facts, and, for this purpose, has drawn up tables of the mortality not only in Prussia, but also in Britain, France, and Belgium.

From these researches he comes to the conclusion, that every where the mortality is directly proportional to the fecundity of the population.

This doctrine, if confirmed by future enquiries, will go far to reconcile the conflicting opinions of Malthus and his opponents; as it shews us that Nature herself tends to remedy the evil of a redundant population.

Dr. C. has given a most valuable table of the mortality in Berlin for twelve years, from 1817 to 1829, which comprises nearly 70,000 deaths in nearly 2,000,000 inhabitants.

The following are a few interesting data which are derivable from his researches.

The longevity of the female is greater than that of the male sex.

The age of puberty carries off 8 per cent. more of the female than of the male sex.

The proportion of deaths of women in labour is 1 in 108.

It has been an erroneous, although hitherto a very prevalent, notion, that the climacteric age of women has a marked influence in increasing the mortality of the female sex.

Two able Frenchmen, MM: Deparcieux and Benoiston de Chauteauneuf, had, several years ago, showed the incorrectness of this idea; and the researches of Dr. Caspar quite confirm the accuracy of their statements. On the whole therefore, we may positively assert that the longevity of the female is greater than that of the male sex.

It is also worthy of being mentioned that, of still-born infants, there are more of the male than of the female sex.

M. Caspar proceeds to show that the medium or average duration of life has increased considerably in most European cities, of late years. In London this increase is immense; for it would seem that, within the last century, '*la vie probable y a augmenté*' by twenty years.

At Geneva, again, in the 16th century one half of the infants born there died, we are told, before their fifth year; whereas, in the present day, it would appear that this half reaches nearly 43 years of age.

A similar remark may be made as to the increased longevity of life at Berlin.

Before the researches of Dr. Caspar, statists had paid little attention to the influence of pursuits and occupations on the duration of human life. He has investigated the subject with great assiduity, and from his enquiries it appears that *clergymen are*, on the whole, *the longest, and medical men are the shortest livers.* The different classes may be arranged, in respect to longevity, as follows:

| | Medium longevity. |
|---|---|
| Clergymen | 65 years. |
| Merchants | 62 — |
| Clerks | 61 — |
| Farmers | 61 — |
| Military men | 59 — |
| Lawyers | 58 — |
| Artists | 57 — |
| Medical men | 56 — |

Another important agent or influence on the probable duration of life is *marriage.*

It is most indubitably proved by the researches of our author that *the married state is favourable to longevity*; and especially in reference to the male sex.

It is almost unnecessary to allude to the influence of poverty and destitution in shortening the medium duration of human life. Dr. Caspar gives some tables of mortality, which prove how sad is the contrast in this respect between the poor and the affluent. From these it would seem that the medium age of the nobility of Germany may be stated to be about 50 years, whereas that of the paupers is as low as 32 years.

The last chapter of the work treats of the influence of the fecundity of a population upon its mortality.

Dr. Caspar shews by a vast number of documents that '*the mortality in any population is always in exact ratio to its fecundicity,*' or, in other words, '*the more prolific that a people is, the greater usually is the mortality among them.*'

He alludes to the difference, in this respect, in the different districts of England; and he proves by numerous data that, wherever the number of births is highest, there the mortality is greatest at the same time.

The same result is derivable from certain statistical investigations in Belgium, France, and other countries.

Dr. Caspar has deduced from the tables in the Annuaire du Bureau des Longitudes, that there is a difference of at least six years in the medium duration of life, in favor of the less over the more populous districts of France.

Dr. Caspar closes his work by embodying the general results of his researches in the following conclusions:

1. The proportion of births to the actual stationary population of any place expresses, or is relative to, the medium duration of life in that population.

2. The female sex enjoys, at every period or epoch of life—except at puberty, at which epoch the mortality is rather greater among young females—a greater longevity than the male sex.

3. Pregnancy and labour occasion indeed a considerable loss of life; but this loss disappears, or is lost, in the general mass.

4. The so-called climacteric periods of life do not seem to have any influence on the longevity of either sex.

5. The medium duration of life, at the present time, is in Russia about 21 years, in Prussia 29, in Switzerland 34, in France 35, in Belgium 36, and in England 38 years.

6. The medium duration of life has, in recent times, increased very greatly in most cities of Europe.

7. In reference to the influence of professions or occupations on life, it seems that ecclesiastics are, on the whole, the longest, and medical men are the shortest livers.

Military men are nearly between the two extremes; but yet, proportionally, they more frequently than others reach very advanced years.

8. The mortality is very generally greater in manufacturing than in agricultural districts.

9. Marriage is decidedly favourable to longevity.

10. The mortality among the poor is always greater than among the wealthier classes.

11. The mortality in a population appears to be always proportionate to its fecundity—as the number of births increases, so does the number of deaths at the same time.

From the preceeding extracts from Dr. Caspar's work, Die Wahrscheinliche Lebensdauer, 8vo. p. 216, Berlin 1835, our readers will be able to judge for themselves of its high value and interest.

---

OF THE INTRODUCTION OF AIR INTO THE VEINS, DURING SURGICAL OPERATIONS.

A Commission was appointed last Autumn by the Royal Academy of Medicine at Paris, to report upon a long and very elaborate memoir presented by M. Amussat, on the 'Introduction of Air into Veins.'

This Commission consisted of seven members—MM. Velpeau, Blandin, Gerdy, Barthelemy, Adelon, Moreau, and Bouillaud—and the report, drawn up by the last of these gentlemen, was recently submitted to the Academy, and excited great and very general interest.

To those, who wish to make themselves acquainted with the literary history, as well as with the opinions of different writers regarding this curious point of physiology, we strongly recommend the perusal of the original report, in the Memoirs of the Academy ; as our very limited space necessarily precludes us from doing more than giving the more general results of the enquiry.

M. Bouillaud alludes in the first place to the interesting speculations of M. Poiseuille and of the late Sir David Barry, on the influence which the respiratory movements of the dilatation and contraction of the chest have on the motion of the blood in the large veins.

He gives an analytic view of the experiments performed in 1811 by M. Nysten on the effects of introduction of air into the veins ;* and proceeds to mention the opinions of M. Magendie on this subject. The last-named physiologist, after stating that the danger in such cases arises from the heart becoming distended with the introduced air, and being thus paralysed, suggests the practice of introducing an elastic-gum tube into the divided vein, and exhausting the air with a syringe affixed to it. "You have," says he, "a ready mode of satisfying yourselves whether any air remains in the heart or not : all that is necessary is to apply the ear over the cardiac region, in order to ascertain if the 'fremissement characteristique' is perceptible."

M. Bouillaud then reviews the various cases on record, in which *it has been alleged* that the entrance of air into the circulation has occurred in the human subject, and gives the details of six of the most interesting. They amount in all to 30.

In 1811, M. Bauchene, while extirpating a large tumor from the shoulder, was surprised to hear a sound altogether similar to that which the air makes, when it enters the chest of a living animal by a small aperture. (It was suspected that the pleura had been wounded.) The patient cried out :—*Mon sang tombe dans mon cœur ; je suis mort.* He died 15 minutes afterwards.

*Dissection.* All the cavities of the heart were empty of blood. The cerebral vessels, the inferior cava, the iliac

---

* The following passage from M. Nysten's work is curious, as alluding to the value of auscultation, before M. Laennec had even dreamed of his future discoveries.

After having suggested that some cases of asthma may depend on the presence of a gaseous fluid, accidentally developed, in the heart, he adds :—

"I am persuaded that, by applying the ear over the region of the heart, we might distinguish, by a peculiar sound, those cases of asthma which are owing to this cause."

veins, the aorta, and the crural arteries contained blood mixed with bubbles of air. The pleura had not been wounded.

2. In 1822, a similar accident occurred in the practice of M. Dupuytren. He was removing a tumor from the neck of a young girl. While engaged in detaching it from its connexions, he heard a prolonged blowing or whistling sound, like that caused by the entrance of air through a small orifice into a vacuum. M. Dupuytren said, *Si nous n'etions aussi loin des voies aeriennes, nous croirions les avoir ouvertes.* The patient exclaimed, "I am dead;" sank down on the chair, and expired.

*Dissection.* The right auricle was found so distended with air, that it gave out an elastic feeling on pressure, and, on dividing its parietes, the air escaped with considerable force. The blood in the bloodvessels, in different parts of the body, contained so much air that when they were pricked, it (the blood), which flowed out, was frothy and bubbling.

3. M. Delpech mentions the case of a man, whose arm he removed at the shoulder-joint. No sooner was the member removed than the patient expired, as if he had been struck with lightning.

We are informed that, on *dissection*, "on vit s'echapper une quantité prodigieuse de bulles d'air, à l'incision des vaisseaux et du cœur."

4. In 1826, M. Castara, while removing a tumor from the shoulder and neck of a young man, heard a peculiar sound, a sort of *glou-glou*, proceeding, as it were, from the bottom of the wound. The patient became instantaneously insensible, drew two or three deep inspirations, and expired.

*Dissection.* The right auricle and ventricle were found distended, elastic, and crepitating on pressure. On dividing them, a small quantity of blood, mixed with numerous bubbles of air, escaped. The superior cava, and the right subclavian vein, were filled with frothy blood.

5. In 1832, a similar case occurred to M. Roux. The operation was the removal of a large tumor from the neck. He had raised it in one hand, in order to divide more easily its subjacent adhesions. At this moment a whistling sound, like that of air entering into a vacuum, was heard; the patient emitted a moaning cry, the breathing became exceedingly distressed, and death seemed to have taken place. Compression was made on the wound, stimulants were administered, and, after some short time, the girl began to revive. She died, however, on the 7th day after the operation.

*Dissection.* The cavities of the heart were empty. The aorta, when pricked at various points, emitted a sanguinolent serosity, which was mixed with numerous bubbles of air; the iliac arteries presented the same phenomenon; but no traces of air were discoverable in any of the veins.

6. In 1836 a patient, whose arm M. Roux was removing at the shoulder-joint, was observed to be unusually pale and death-like. In spite of every means of resuscitation, he expired.

*Dissection.* The right ventricle was found to present an unusually elastic distension on touch. The coronary veins contained numerous globules of gas; and the same phenomenon was observed in the vena cava.

Within the last few months M. Amussat has communicated another case of a similar accident—only that the patient survived; a fortunate result, which M. Amussat attributes to the forcible compression of the chest which was resorted to.

Such are the cases, which are alluded to by M. Bouillaud as having occurred in the human subject, during the performance of operations.*

---

* Since the publication of M. Bouillaud's report, M. Velpeau, one of the Commission, has communicated to the Gazette Medicale a very elaborate notice of all the cases, real or alleged, which have hitherto been recorded.

We shall give an abstract of his paper in our next number.—*Rev.*

With the view of throwing some light on this curious point of pathology, M. Amussat has performed a great number of experiments on dogs and horses, to ascertain whether the phenomena, which have been attributed to the entrance of air into the divided veins, ever occur in the lower animals; and in what manner they are affected, when such is the case.

These experiments, amounting in number to 40, were performed before the members of the Commission. In some, the divided veins were not disturbed; in others, they were kept patent, so as to favour the admission of air along their tubes; and in a few instances, the air was introduced either by means of a syringe, or by insufflation with the mouth.

The general results of these experiments we shall now attempt to submit to our readers' attention.

M. Bouillaud observes:—

"Every time that one of the jugular veins has been opened, by a sufficiently large incision, in its lower portion—where the phenomena of a flux and reflux, isochronous with the movements of inspiration and of expiration, are observable—a certain quantity of atmospheric air immediately enters, and penetrates into the right cavities of the heart.

The indicative, and in some degree pathognomonic, sign of this accident, is a peculiar sound which resembles that of lapping, or gurgling, and may perhaps be best expressed by the word *glou-glou*.

It is evident that its *principal cause* is the expansion of the chest during inspiration; the sound, both in quickness of succession and in loudness, distinctly corresponding with the frequency and vigour of the inspiratory movements.

The dilatation and contraction of the right ventricle and auricle of the heart may be mentioned as a *secondary* or *adjuvant* cause.*

In addition to the *external* sound, we may very generally perceive an *internal* or thoracic one, by applying the ear over the cardiac region. It is a sort of *blowing* or *gurgling sound*, like that caused by stirring water and the white of eggs together.

We have already stated that several physiologists—more especially M. Poiseuille and Sir David Barry—had, some years ago, drawn our attention to the influence of the respiratory movements on the current of the blood in the large veins adjacent to the heart; but it is to M. Amussat, says the reporter, that we owe the clear elucidation of the fact, that *whenever a sufficiently large opening is made in one of the jugular veins*—where the movements of flux and reflux are observable—*the atmospheric air invariably enters into the sanguineous system.*

The same occurrence is apt to happen when the subclavian or axillary vein is divided; as we find was the case in some of the instances in the human subject, recorded above.

It will now be useful to mention briefly the most interesting results of M. Amussat's experiments on the lower animals.

In 15 of the dogs, which were examined immediately after having been submitted to the spontaneous entrance of air into a divided vein, a greater or less quantity of air was discovered within the right cavities of the heart and in the pulmonary artery. The distention of the right auricle and ventricle, thus induced, was in some instances so great, that these cavities had acquired three times their normal size. This *ballonnement*, says M. Bouillaud, is a constant phenomenon.

In some of the animals, air was found mixed with the contained blood, in the facial and cerebral veins also. In several dogs, which did not die for several days after the introduction of air into their veins, bubbles of air were found on dissection at various points of the vascular system; sometimes in the left

* M. Bouillaud says:—

"The phenomenon of the flux and reflux of the blood in the lower part of the jugular veins takes place sometimes under the influence of the alternate movements of the right cavities of the heart, as well as under the influence of inspiration and expiration, as noticed above."

cavities of the heart and in several of the arteries, and at other times chiefly in the right cavities and in the veins. We may therefore conclude that, if an animal survives for some time the entrance of air into a divided vein, it is propelled along the pulmonary artery into the corresponding veins, and thence conveyed into the systemic circulation.

Hitherto physiologists have too generally assumed the idea that very sudden, or rather almost instantaneous, death follows the admission of air into the sanguiferous system.

In the case of the lower animals, at least, such is certainly not the case; and there is also good reason to extend this observation to the human subject: as several patients have recovered after operations, in which the peculiar gurgling sound, indicative of the entrance of air into the veins, has been heard.

M. Bouillaud frankly acknowledges that he had expected to find the mortal effects of the accident to be much more rapid, in the lower animals, than proved to be the case on experiment.

In all however, after a lapse of a very short space, varying from one to ten minutes, certain phenomena were observable. These were a rapidity and embarrassment of the breathing and of the circulation, an exhaustion of the general strength, and an anxiety and restlessness of the whole body.

If the experiment closed on the development of these symptoms, many of the animals quickly recovered a state of ease, and gradually recovered.

If, on the contrary, the introduction of the air continued, the distress of the animal gradually increased, the breathing became more hurried and oppressed, the exhaustion greater, and, after a few convulsive movements, life was extinguished.

The rapidity of the death—varying, though it does, according to the size and strength of the animal, its state of health before the experiment, the quantity of air admitted, and the quickness with which it entered—may be judged of from the following particulars.

Out of fifteen dogs, eight died in from a few minutes to half an hour after the introduction of the air.

It was observed that, if the animals were sickly or weakened from loss of blood, at the time of the experiment, the fatal effects of the accident were considerably more rapid.

Again; in eight horses, subjected to the experiment of the spontaneous introduction of air into the blood, from an incision being made in one of the jugular veins, death took place in from nine minutes to an hour and a half—the death being much more rapid in those animals which were diseased or exhausted from loss of blood than in such as were not, at the time that the experiment was performed.*

Before quitting this subject, it is right to mention that the rapidity, with which death takes place, is infinitely greater when the air is blown into the vein from the mouth of the experimenter, than when it enters spontaneously from the atmosphere.

Three dogs died, in the course of from half a minute to two minutes, by a single insufflation; and three horses were killed, in from four to six minutes, by two insufflations.

In this mode of the introduction of air into the veins, the animals usually die, as if they were struck with lightning, or felled with a blow.

Whether the extreme rapidity of the death, under such circumstances, is attributable altogether to the force with which the air is introduced into the sanguiferous system; or whether it is partially owing to the vitiated gas which is admitted, must be determined by future experiments.

If we now enquire into the probable causes of death, when air enters the sanguiferous system, M. Bouillaud directs our attention to the following three:

1. The enormous distention of the right cavities of the heart, inducing an irregularity, or even a complete cessation, of its movements.

---

* It deserves to be noticed that the period of time, at which death usually happened after the experiments alluded to, varied much less in the horse than in the dog.

2. The presence of air in the pulmonary artery and in its branches, causing an embarrassment of the pulmonary circulation, and perhaps also a contraction of the aerial cells.

3. Compression of the brain, from the air being occasionally admitted into the cerebral vessels.*

Such, in the opinion of M. Bouillaud, is the *mechanism of death,* when the introduction of air into the veins proves fatal.

In conclusion, the learned reporter observes, that the results of M. Amussat's experiments on dogs and horses, as well as the phenomena which were remarked in the six cases in the human subject, (alluded to before), leave no doubt on his mind that air may enter any divided large vein about the upper part of the chest, and, by being carried on to the heart, induce a speedy death.

But whether the almost momentary or instantaneous rapidity of the death, which has occurred in some instances in the human subject, is to be attributable solely to this cause, seems still somewhat doubtful. At least, in none of M. Amussat's experiments did the *spontaneous* introduction of air prove fatal to either dogs or horses in less than nine or ten minutes. It may therefore be fairly presumed that the mental alarm of a human being at the moment, not to mention the unhealthy or enfeebled state of his body at the same time, may tend much to accelerate the fatal event. —*Memoires de l'Academie,* 1837.

*Remarks.*—In our next number we propose to continue the investigation of this very interesting subject, and give a brief summary of the opinions of the leading surgical members of the French Academy, as expressed in their comments on the report of M. Bouillaud. —*Rev.*

---

* It is worthy of notice here, that in all the horses, which perished from the spontaneous introduction of air into the blood in M. Amussat's experiments, air was found in the cerebral vessels on dissection; a phenomenon which was of comparatively rare occurrence in the dogs experimented upon.

### ON THE RADICAL CURE OF HERNIÆ BY ACUPUNCTURATION.

M. Bonnet, one of the surgeons of the Hôtel Dieu at Lyons, has contributed a very valuable paper to the Gazette Medicale on the above subject. He has been prosecuting his enquiries for several years past, and in that time he has met with so many cases, which fully warrant him in recommending the practice alluded to, that he is now anxious to communicate his experience to the profession at large.

The method, which he employs, is to insert several needles near to the inguinal aperture and fairly through the herniary envelopes, and then, after arranging them so that the opposite parietes of the sac are brought and kept in contact, to leave them in this situation until adhesive inflammation has been established.

Before detailing particular cases to illustrate the effects of this practice, M. Bonnet alludes to several applications of this form of acupuncturation in surgery. MM. Velpeau and Carron du Villards have, for example, used it for the purpose of inducing obliteration of arteries; M. Duvat in the treatment of varicose veins, and several other surgeons in cases of hydrocele, of encysted watery tumors, and of nævi, or aneurisms by anastomosis.

But it is unnecessary to do more than simply to call the attention of our readers to these particulars, without entering upon any of their details. The object in all these cases is to excite the effusion of lymph, and thus to induce an agglutination of parts previously apart.

The method, which M. Bonnet adopts in the treatment of herniæ, is as follows.

Having reduced the protruded bowel, he lays hold of the sac close up to the ring, keeping the spermatic cord fairly and firmly to the outside of the forefinger and thumb of his left hand: he then passes a long needle quite through the entire thickness of the part, guarding the head and point of the instrument, when it is fairly through, with pieces of cork, so as to prevent its displacement. Sometimes he twists or curves the two ends of the needle, as a further security against its slipping out.

He afterwards passes several other needles, with similar precautions, on the inside of this one; and sometimes also one or two on the outside of the spermatic cord. The number of needles that he usually employs is from six to eight. It is well to pass a thread through one of the pieces of cork affixed to each needle, for the purpose of aiding its withdrawal, in case it be imbedded in the swollen part.

The needles are to be withdrawn on from the sixth to the twelfth day; and then a poultice or a compress, wetted with some spirituous wash, should be applied. As soon as the tenderness of the part abates, a compressive bandage is to be used.

The following is the catalogue of cases treated in this manner by M. Bonnet.

*Case* 1. A man, 66 years of age, had been afflicted with an inguinal hernia on the right side for seven years. The inguinal canal was so wide that two fingers might readily be passed along it; and hence the protruded mass was as large and bulky, as two fists when closed together.

Six needles were introduced in two rows, one above or nearer to the inguinal aperture than the other.

The inflammatory action in old people being tardy, the first needle was not removed till the eighth day after insertion; a second on the thirteenth, and the rest on the following two days. The patient kept his bed for ten days; after this time he rose; and it was then found that the cough, which always caused the rupture to descend quickly before the operation, had no effect upon it.

A fortnight later he was dismissed, apparently cured, but wearing a truss.

It seems, however, that the old man's cough continued afterwards to be as troublesome as ever, and that, in consequence of the continual succussion thus kept up, the rupture came down again three months after he left the hospital.

*Remark.* It is to be noticed that everything was unfavorable to a cure in this case—the age of the patient, the size of the hernia, and the constant cough.

*Case* 2. An idiot, 45 years of age, had been ruptured for twelve years in the left groin, when M. Bonnet saw him. He would never wear a truss; so that every now and then the hernia became exceedingly large, descending as low as the middle of the thigh, very painful, and almost irreducible: the inguinal aperture was so wide, that three closed fingers might be passed through it.

Nine needles were introduced, four in one row, and five in the other.

For the first six days, there was but little pain or irritation. After this period, however, the sac began to swell, and a blush of redness surrounded the seat of the punctures.

By the twelfth day, the punctures were found to be ulcerated; the needles, therefore, were withdrawn on this and on the following day.

On the 17th day, the herniary sac was felt to be empty; but the inguinal aperture was still perceptible, although it was much less open than before.

Unfortunately, at this period, an attack of erysipelas came on, accompanied with a troublesome cough. The result of this was the renewal of the herniary protrusion.

3. The following case was more successful.

A locksmith, 38 years of age, had been afflicted with an inguinal rupture for 13 years: it was as large as the closed fist. M. Bonnet passed four needles through the sac, as near to the ring as possible. For the first four days, the patient experienced but little inconvenience; but then the seat of the punctures became red and painful; and by the sixth day the punctures were more or less deeply ulcerated. The needles were removed on the following day.

When the tenderness, &c. had subsided, the patient renewed the use of a truss; but every now and then he laid it aside: yet it was found that, even when a fit of coughing came on, there was no return of the herniary swelling. M. Bonnet saw him at various intervals during the next twelve months, and satisfied himself repeatedly that the inguinal aperture had become nearly, if not quite, impervious.

The *fourth* case occurred in a young man, 24 years of age: the hernia was not larger than a hen's egg, and it did not protrude, except after exertion or coughing. He had been recommended to wear a truss, but finding the use of it to be very inconvenient while engaged at work, he had laid it aside.

This therefore was a favourable case; and the result of the acupuncturation corresponded with the hopes of M. Bonnet. Three needles only were introduced: on the eighth day they were withdrawn. He remained in the hospital for another fortnight, and was then discharged.

M. Bonnet saw him repeatedly during the next fifteen months, and satisfied himself of the permanence of the cure.

In the *fifth* case, the hernia was of seven years' standing: the patient was 23 years of age, and the tumor was as large as the fist. Five needles were passed: they were removed on the seventh and eighth days.

It is only necessary to add, that there was never any threatening of the protrusion afterwards. The patient however continued the use of a truss, every now and then, with the view of consolidating the cure.

M. Bonnet alludes to two or three other cases, and also to an instance of umbilical rupture in a dog cured by his method; but our space prevents us from giving their details. It is important however to add, that he has had two opportunities of examining the state of the parts after the operation—in one case, 21 days after the withdrawal of the needles; and, in the other, a year and a half after the date of the operation.

In the first of these examples, the patient died from an apparently aggravated form of suppurative or rheumatic fever. A large abscess in the psoas muscle was found on dissection, and several of the abdominal viscera exhibited marks of disease.

The subcutaneous cellular substance at the seat of the acupuncturation was thickened and infiltrated. The fibrous membrane enveloping the herniary sac, and continuous with the aponeurosis of the *obliquus externus* muscle, did not exhibit any appearances of change. One only of the five needles had passed fairly through this envelope; the other four *avaient passé en de hors de sa cavité.*

On slitting open the peritoneal sac, its anterior and posterior surfaces did not adhere, except at one point only, where a fibrous column, two lines in length and as thick as a writing-quill, connected these surfaces together; thus dividing the peritoneal sac into an inner and an outer canal.

*Remark.* The unsuccessful issue of the preceding case was unquestionably attributable to the visceral and other disease, which was existing at the date of the operation. It cannot therefore be fairly deemed as arguing against the propriety of the practice.

The other case, in which M. Bonnet had an opportunity of examining the state of the herniary tumor after the operation by dissection, occurred in an idiot 49 years of age. He had been affected with rupture from his infancy. The tumor was so large that it hung down to the middle of the thigh, and the inguinal canal was wide enough to allow the passage of four closed fingers.

M. Bonnet passed eight needles; but there was great difficulty experienced in retaining them in their situation. Considerable swelling and tenderness of the sac supervened, and a considerable effusion of fluid into its cavity took place. This however subsided by rest and the use of discutient applications. By the end of the fourth week, the patient was permitted to rise from bed; and it was found that even on coughing the herniary tumor did not protrude. It seems however that, two or three months after his discharge from the hospital, the rupture re-appeared. He returned to the hospital about a twelvemonth afterwards, in consequence of a thoracic affection, which ultimately proved fatal.

On the dissection of the herniary tumor, M. Bonnet found three small fibrous cords, each of about the size of a writing-quill, which bound closely together the peritoneal sac and its outer fibrous envelope.

M. Bonnet supposes that there were similar connecting cords, connecting the anterior and posterior surfaces of the sac itself, and that these were probably ruptured, when the bowels protruded two or three months after the operation.

It is worthy of notice that, before dissecting the parts, M. Bonnet passed eight needles through the neck of the herniary sac, in order to ascertain what immediate effects were produced by the operation. It was found on examination, that *three only had traversed the sac.* There is therefore, it would seem, considerable uncertainty attending the use of acupuncturation, as described above.—*Gazette Medicale.*

---

### Cases of Phlebitis after Amputation.

The accident of inflammation of the divided veins after amputations of the limbs is always very serious and alarming. Most cases of internal, or interstitial, abscesses are found to be connected with this morbid state of the venous tubes. The pathology of this affection will be illustrated by the following details.

1. M. Blandin, in 1830, amputated the leg of a middle-aged man, in consequence of a cancerous growth upon it. Every thing went on favourably for the first week; at the end of which time however, the patient had a shivering fit, which ushered in an attack of pleurodynia. This subsided; but, a week subsequently, a second attack was experienced; and the wound, which hitherto had been going on well, began now to assume an unhealthy aspect, the granulations being very pale, and the suppuration thin and scanty.

Typhoid debility, attended with diarrhœa, delirium, &c. came on, and the patient died at the end of the third week after the amputation.

*Dissection.* Numerous minute vomicæ were found dispersed on the surface of the left lung, more especially in its inferior lobe. The liver and spleen were healthy.

The popliteal artery, for some inches above the stump, was thickened in its parietes, and filled with purulent matter: several of the muscular branches, terminating in it, exhibited similar appearances. The femoral vein was not affected. Pus was found however in the lumbar, and also in some of the pelvic, veins.

2. A man, 60 years of age, met with a compound fracture of the leg, which M. Breschet deemed to call for amputation. No fewer than twelve ligatures were necessary.

Two or three days afterwards, the patient became agitated and feverish, and the wound began to emit a fetid sanious discharge. By the end of the second week, the patient was exceedingly reduced in consequence of a troublesome diarrhœa, and also of hectic symptoms having supervened. He became delirious, comatose, &c. and died on the 18th day after the operation.

*Dissection.* A minute abscess was found in one of the convolutions of the right cerebral hemisphere: another, somewhat larger, was detected in the cerebellum.

A purulent effusion was present in the right pleural cavity; and ten or twelve abscesses, more or less imperfectly developed, were scattered through the pulmonary substance, especially in the lower lobes.

Two small abscesses were found in the liver, under its peritoneal coat.

On examining the stump, the divided bones* were found bathed in pus; and,

---

* In a short article on phlebitis in the Foreign Periscope of our last number, M. Duplay refers very pointedly to the frequent infiltration with purulent matter of the divided bones after amputation, and to the *inflamed state of the osseous veins* in cases of secondary phlebitis.

on sawing them across longitudinally, their spongy texture was observed to be quite infiltrated with purulent matter, which extended upwards to nearly the articular cartilages.

*All that is mentioned of the state of the veins* is simply that they were *blanchatres et obliterées.*

3. A young man had to submit to amputation of the thigh, in consequence of white-swelling of the knee-joint. This was on the 26th of December. For several days afterwards he was restless and feverish; the wound did not unite but suppurated freely and unhealthily. Secondary hæmorrhage came on on the 7th of January, and he sunk next day and died.

In addition to an extensive sinus which extended from the pelvis to the stump, the veins of the amputated extremity were found to be diseased. In the internal iliac, and more abundantly in the crural, vein, purulent matter mixed with blood was found; and the internal membrane of these vessels was thickened, red, and easily lacerable.

4. A young man underwent amputation of the thigh for a diseased knee-joint.

On the third day after the operation, he was seized with chills and feverish symptoms. In the course of a fortnight afterwards, an abscess formed over the sacrum; the patient became weaker and weaker, and died 23 days after the removal of the limb.

*Dissection.*—A deep sinuous abscess, extending from the face of the stump along the line of the crural vessels as high as the groin, was detected.

The crural vein contained purulent matter and membranous deposits on its inner surface, which was of a greyish black colour.

Several small abscesses were found in different parts of the lungs; and in several of the joints a semi-purulent effusion was discovered.

---

It is therefore important that surgeons attend in future to this point in the pathology of the disease.

5. In this case, the crural vein, on the face of the stump, was found gaping, and filled with an admixture of pus and dark blood: its inner surface was coated with a membranous deposit. Similar appearances were discovered in the common iliac vein, and also in the lower extremity of the vena cava.

Numerous circumscribed abscesses, more or less partially formed, were seen in different parts of both lungs.

For the details of the other cases reported, or alluded to, by M. Duplay, we must refer to his memoir.

He has carefully analysed the particulars of 25 cases, related in different surgical works; and from these researches he has deduced the following, among some other, conclusions.

Of 23 cases—in two the examination of the internal viscera did not take place—vomicæ or abscesses in the *lungs* were found in 19. In 14 of these 19 cases, the abscesses occupied both lungs.

Secondary abscess of the *liver* is of much less frequent occurrence: it existed in 3 cases only: in 2 others there were the marks of incipient change, probably preparatory to the suppurative action.

In 2 cases, abscesses were detected in the *spleen.* In 3 cases, there was an abscess in the *cerebrum* or *cerebellum.* In 5 cases, there was purulent effusion into the *joints;* and, in 4 cases, abscesses had formed in the *cellular* substance of different parts of the body.—*L'Experience.*

---

### On the Efficacy of Extension, Shampooing, and Percussion in Muscular Contractions. By Dr. Recamier, of the Hôtel Dieu.

The peculiar functions of all the organs of the body may be disturbed, either directly or indirectly—the deviations from health being in many cases dependent upon the state of an organ at a distance from the one, which exhibits the morbid phenomena.

The contractile functions of the muscles, involuntary as well as voluntary, not unfrequently exhibit the truth of this remark. My object in adducing the following cases is to point out the great efficacy of simple mechanical means in rectifying many muscular ailments.

1. About eighteen years ago, I was consulted by a middle-aged gentleman, who had for upwards of four years been regularly nailed to his bed by a pain in the right side of the neck, shoulder, and arm, which was so agonizing on the slightest movement that often he could not refrain from screaming out.

All sorts of anodynes and emollients had been ineffectually tried. I advised the use of regulated and, as it were, elastic percussion with the hand on the affected part. At first it was to be administered in gentle taps, and gradually with more and more forcible beats. By the aid of this simple means alone, the patient was speedily relieved from all his suffering, and was soon enabled to resume his duties as a judge of the peace.

2. A young girl, 18 years of age, was admitted, in 1836, into the Hôtel Dieu, under the care of M. Recamier, for pleurisy. At the beginning of the following year, the catamenia, which for some time back had been scanty, were suddenly arrested by exposure to wet and cold. Dyspnœa and general *malaise* were the consequence. She was re-admitted into the Hôtel Dieu. Leeches were applied repeatedly to the vulva; but then the left arm, fore-arm, thigh and leg, became affected with extreme rigidity, and these symptoms were accompanied with retention of urine, and with a most painful difficulty in evacuating the bowels.

Bleeding, anodynes, in enemata, as well as given by the mouth, &c., &c., were freely employed, but with no avail; and this poor creature remained for two entire months in this most distressing situation of stiffness of the left limbs and retention of (or rather, we presume, difficulty in passing) the urine and the fæces.

On examining the rectum it was found to be quite empty; but the *sphincter ani* was exceedingly contracted.

I dilated the stricture; the pain, although severe at the time, ceased almost immediately, and the evacuation of the bowels became at once much more easy.

The result of this first trial induced me to treat the urinary affection in a similar manner; and with this view I began to work or knead (*masser)* the neck of the bladder against the pubes, by means of a finger introduced into the rectum; the strangury ceased as quickly as the constipation had done.

I then resolved to apply a similar mode of treatment to the contracted muscles of the limbs, acting as in cases of ordinary cramp.

I commenced with the arm; and it was not without great difficulty that I overcame, little by little, sometimes by continued efforts and at other times by efforts *en cadence*, the resistance of the extensor muscles of the fore-arm, the flexors of the hand, and also of the deltoid and other muscles of the shoulder-joint.

By dint of patience, however, the arm was bent, the hand opened, and the arm was gently removed from the side: then laying hold of the hand, I kept swinging it about.

After this rather violent, and moreover painful, manipulation had been continued for some time, it was found that the patient was able to move her arm about herself.

My attention was then directed to the affected lower extremity; but here it required the strength of three people to bend first the knee and then the thigh on the pelvis. We may readily suppose that these efforts were attended with great suffering to the patient. When the pliability was once restored, the limb was swung about from side to side for some time. This did not require to be continued long, till the girl was able to use the limb herself, so as to walk about the ward.

Gradually she recovered the use of all the contracted parts, so that she soon left the hospital nearly quite well.

The *third* and *fourth* cases were of wry-neck; in both, the disease was cured by the gradual but forcible extension of the contracted muscles.

The *eleventh* case which M. Recamier relates, was one of permanent and painful rigidity of the muscles on the back of the neck, in an elderly lady, who was in consequence of the affection confined constantly to her sofa. A variety of means had been ineffectually tried for a length of time, when, on the suggestion of our author, shampooing and compression speedily effected a cure.

5. Five and twenty years ago, I was consulted by a lady, who had long severely suffered from a *fissure of the rectum,* for which the late Baron Boyer had divided the sphincter.

The operation however, did not prevent a relapse of the disease, and the patient continued to suffer dreadful pain in the rectum, especially on going to stool.

Dilatation of the sphincter and of the lower end of the rectum by means of bougies, gradually increased in size, ultimately succeeded in effecting a perfect cure.

It is to be remarked that in this, and in many other similar cases, the dilatation was attended with excruciating pain.

Dr. Recamier mentions several other instances of painful constriction of the anus, either simple or complicated with hæmorrhoids, fissures of the rectum, &c., in which the use of gradual but forcible distention of the gut was speedily followed by great relief, and ultimately by complete recovery.

He adds that the surgical operation of dividing the sphincter may be dispensed with in the majority of cases.

6. Several years ago I was sent for to a middle-aged lady, who was suffering dreadful torture from *une colique nerveuse et apyretique.*

Without delay I placed my extended palms on her belly, and commenced a gradual and firm compression—this had not been continued long before the severity of the suffering quite ceased.

In a future paroxysm I made her waiting-maid *asseoir sur le ventre de sa maitresse;* and this mode of compression was speedily effectual (!)

In another case of a like nature, I have used with success a binder drawn very tightly round the abdomen; adding, if necessary, a pad or cushion over the seat of the pain at the same time.

When in severe colic the intestines are felt through the abdominal parietes, like hard cords or serpents, I have repeatedly relieved the patient's sufferings by kneading them, as it were, with my hands, so as to overcome their unnatural state of constriction.

7. A lady, thirty-two years of age, had long suffered from excruciating pains in the hypogastrium, unattended, however, with fever. On examining per vaginam, the uterus was felt to be quite healthy; but, on examination by the rectum, the posterior surface of the uterus was found to present several inequalities to the finger. This case I regarded to be one of a purely nervous, or muscular, character, and likely therefore to be benefited by compression.

Grasping the uterus in the hypogastium with my left hand, I pressed upon, with two fingers of my right hand in the rectum, the inequalities just now mentioned; I was surprised to find them gradually disappear; while at the same time the patient, who at the beginning of the experiment suffered most severe pain, declared that she was now comparatively quite easy.

These *bosselures* having been in this manner dissipated, on three or four occasions, the pains ceased to return.

The cure was rendered permanent by the use of a bandage, tightly laced round the pelvis and hypogastrium.

What relation is there, adds M. Recamier, between the cause of these uterine pains and the partial spasms of the womb—an organ which we know to be muscular and eminently contractile?

The next two cases, which our author records, are instances of violent intestinal spasms, which were speedily relieved by the employment of forcible compression, and of enemata of warm water administered at the same time.

10. A lady, thirty years of age, had for several months been affected with a permanent and apyretic hiccup, the *secousses* of which were so violent, as quite to lift up and shake every part of the body. Various remedies of an anodyne, antispasmodic, &c., nature had been employed, but without decided benefit. I suggested the use of a firm belt, provided with a pad or cushion, placed over the pit of the stomach. By this simple means alone, the patient got entirely rid of her annoying ailment.

12. A young lady was, in 1834, affected with various chlorotic symptoms, for which steel, active exercise in the open air, &c. &c. were recommended by Dr. Colson of Beavais. In 1835, after exposure to wet and cold, she began to be affected with an incurvation of the spine to the left side, so that the trunk formed at length an angle of 45 degrees with the vertical axis of the pelvis; and, at the same time, the right shoulder was lifted up to almost a level with the head, and the right forearm was immoveably contracted upon the arm. Such was the condition of this poor invalid, when she was sent to Paris to be seen by MM. Andral and Marjolin and by myself.

Leeches, cupping over the spine, baths, &c. had been repeatedly tried; but without any effect. The result of the metropolitan consultation was to recommend the use of gymnastic treatment, of fumigations, of leeching and cupping, of embrocations, &c.—but these means were used with no better results than heretofore.

She was then put under the care of M. Guerin, and subsequently of M. Humbert at Morlaix; and, although nearly two years were spent in trying various remedies, the condition of the patient was little, if at all, improved.

Upon her return from Morlaix, she once more consulted M. Recamier, who, remembering the striking results obtained in the second case, suggested to M. Colson to try a similar mode of treament.

Severe pain was caused by the forcible extension of the fore-arm, combined with the shampooing of the biceps muscle. This might have been expected, seeing that the muscle had been permanently contracted for upwards of three years.

The gradual extension and kneading of the muscles of the affected shoulder and of the trunk were attended with much less pain; indeed the manipulation, although very irksome, was almost immediately followed by a feeling of great relief. The improvement of the state of the shoulder and of the affected side of the neck was speedily most remarkable; the condition of the arm and fore-arm was not so promising.

By continuing, however, steadily the same plan of treatment, this young lady gradually recovered the use of the contracted limbs, and was enabled to resume *ses anciennes habitudes;* whereas, during the preceding three years, she had been quite shut out from society, and an object of great helplessness.

13. Last December I was sent for to meet MM. Lisfranc and Chevreux in consultation upon the case of a middle-aged lady, who had been affected some months previously with hysterical ailments; on the cessation of which there supervened a violent pain, first in the coccygeal and then in the cervical and occipital regions, recurring in fits of the most excruciating agony.

During the continuance of these most severe sufferings, there were also, now and then, symptoms of a subacute inflammation of the uterus and its appendages present.

The patient had been visited by M. M. Andral and Chomel. A host of medicines, antiphlogistic, anodyne, epispastic, derivative, &c. had been tried; but without any decided benefit. The quantity of opium, which she had taken without producing even narcotism, was immense. What had procured perhaps more relief than anything else was the application of four grains of the extract of stramonium to a blistered spot on the scalp; but the symptoms of poisoning from it were so alarming that the physician was unwilling to repeat the remedy.

M. Recamier, having attentively studied all the phenomena of this very aggravated case, suggested the following means to be tried:—a firm belt round the hypogastrium, provided with a strap and cushion to compress the os coccyx and the fundament; enemata of assafœtida, camphor, castor, opium, and sometimes of quinine, when the return of the paroxysm appeared to be at all periodic; the internal use of pills of musk, camphor, and assafœtida; electro-puncturation; and lastly the extension and shampooing of all the muscles, which were at any time affected with cramp.

Before leaving the house of the patient, M. Recamier had an opportunity of witnessing one of her dreadful paroxysms of pain, which had, hitherto, usually lasted for three or four hours: the head was thrown backwards, and her features were distorted by convulsions. Having satisfied himself that the muscles on the back of the neck and shoulder were violently contracted, he requested MM. Lisfranc and Chevreux to fix the two shoulders, while with one hand he (M. Recamier) forcibly drew the head forward, and with the other he *kneaded* and *pommelled* the affected muscles.

The patient all this time was screaming out with pain; but no sooner was the head fairly bent forward than she began to smile, and confessed that she was quite easy. M. Recamier advised that the head should be moved about from side to side for some time, in order to prevent the speedy return of the cramp.

The future relapses of the disease were always treated in the same simple manner, and with equally gratifying success.

(The report stops here.)

M. Recamier closes his interesting and instructive paper by the enunciation of the following conclusions:—

*a.* It is very necessary to discriminate those spasms or contractions, which are not dependent upon, or proceed from, an affection of the nervous system, but which constitute a direct loss of the contractile functions of the affected muscles themselves.

*b.* In idiopathic muscular contractions, in wry-neck, in spasmodic colic, in permanent spasms of the sphincters, &c., the use of extension, compression, and shampooing, and the application of the cupping-glasses, seem to be by far the most efficacious means of treatment.

*c.* Hence it is rarely necessary to have recourse to section of the contracted muscles in such cases. Where we know, or have reason to suspect, that there is an actual degeneration or morbid change of structure in the part, such an operation will probably be necessary.—*Revue Medicale.*

*Remarks.*—We quite agree with the Editor of the French Journal, in which M. Recamier's paper appears, that it is one of the most practically valuable which has been communicated to the profession for a great length of time.

The character of M. Recamier stands very high among the ablest physicians of the French metropolis: from our own experience of the French medical literature, we should be inclined to award the palm of pre-eminence to him and to M. Andral, as the two men of the largest and most comprehensive minds, and the best imbued with the genuine spirit of true philosophy.

Their views on medical questions are always clear and sagacious, and their practice seems to be invariably simple and natural.

They are the very antipodes of such men, (able though these are) as MM. Bouillaud, Velpeau, &c. who too often allow themselves to adopt certain notions of disease, and strive to confine nature within definite and arithmetical calculations.—*Rev.*

---

### PROTO-IODURET OF IRON IN SYPHILIS.

This chalybeate has been of late employed with very decided advantage, internally as well as externally, in the treatment of old syphilitic cases, more especially in persons of a scrofulous or lymphatic constitution. It may be usefully combined with bitters, anti-scorbutics, &c. in the dose of from six to forty grains daily.

As an external remedy it may be employed as a lotion or injection.

The proto-ioduret of iron is obtained by heating together about fifty parts of iodine, and fifteen parts of iron filings, with a hundred parts of distilled water: the liquor acquires a greenish colour. It is to be filtered, and quickly evaporated to dryness, protecting it all the while as much as possible from contact with the atmospheric air.

The salt is to be kept in a firmly corked phial.—*Revue Medicale.*

---

### Hooping-cough treated with Carbonate of Iron.

Dr. Steymann, a correspondent of one of the German journals, very strongly recommends the use of chalybeate medicines in the cold or chronic stage of hooping-cough.

He gives it, at first, in minute doses frequently repeated, alone or in combination with sugar. He always premises the use of an emetic.

*Case* 1. Henry Schraeder, 11 years of age, had been suffering from severe hooping-cough for upwards of two months, when Dr. S. visited him. He prescribed—

Carbonate of iron .. 25 grains,
White sugar ...... 20 grains, divided into ten powders, and one given every three or four hours.

In the course of a very few days there was a marked mitigation of the cough. The quantity of the carbonate in the powders was gradually increased; the hooping ceased, and the patient quickly recovered his strength and plumpness.

*Case* 2. Jules Etier, five years of age, had just recovered from the small-pox, when he was seized with hooping-cough. He had been suffering from most violent paroxysms of it for upwards of three weeks, when Dr. S. ordered him the use of the carbonate of iron. At the expiry of four days, the child was completely relieved from the cough.—*Medicinisches Corresp. blatt.*

N.B. The suggestions in the preceding communication appear to us to merit the attention of medical men.

---

### On the Morbid Diatheses which have successively affected the Nations of Europe.

Dr. Hecker (the well-known author, we presume, of several works on the diseases of the middle ages) selected the above subject as the theme of his discourse last year before the Royal School of Surgery at Berlin.

On a former occasion he had directed the attention of his audience to the general constitutions or prevailing types of epidemic *febrile* disorders, which have spread over Europe in different seasons, and the prevalence of which may be said to characterise different portions of its history. These, he shewed, had varied so much in their leading features during different epidemics, that it is quite impossible for any one to deny the existence of certain *pathological periods or epochs* distinguishable, the one from the other, by peculiar characters.

In the present discourse, he limits his remarks to a few of the *apyrectic* or *non-febrile diseases,* which have prevailed at different periods of the Christian æra; and of these he selects Gout, (we suppose under this term rheumatism also is included,) the Oriental Lepra, Scorbutus, Syphilis, and Scrofula.

The nations of Europe have long suffered, and still suffer, from these disorders. It is to be remarked, however, that they have never prevailed extensively at one and the same period of time; and that their succession has presented some curious and interesting characters. When, and under what circumstances, each made its first appearance, is a subject which, we believe, will never be cleared up. There is indeed strong presumption to believe that some of them, at least, were not known to the ancients; others, and more especially the Gout, (including as we have previously suggested, the varieties of rheumatism,) seem to have been even more widely diffused, and also more obstinate and intractable in its nature, than in the present day. We are led from various sources to suppose that it frequently prevailed over immense extents of country as a general epidemic; just as we see to be the case, in our own times, with influenza, and the class of eruptive fevers. It would indeed be difficult, says Dr. Hecker, to fix, with any degree of precision, the epoch at which this epidemic commenced or ceased; but it seems to be very probable that the date of its outbreak was about two hundred years before the Christian æra, and that it continued to recur, at various intervals, for the next eight centuries.

Several epidemic diseases seem to have succeeded to this one. Of these by far the most important was the Lepra of the East. It was carried into Italy after the conquest of the kingdom of Pontus; but it does not appear that it took root *d'une maniere definitive* in Europe till the second century of the Christian æra, nor did it prevail as a wide-spread epidemic till the commencement of the seventh century.

From this period it spread widely, carrying alarm and misery into all classes and grades of the people. The history of this epoch of Europe is full of the most appalling details of the pestilence. Vast lazarettoes were established in every country for the seclusion of the lepers, who were not only deprived of their freedom, but also declared incapable of retaining their civil or personal privileges.

In the thirteenth century, France alone had upwards of 2000 of these leper-houses; and in the other nations of Europe there were more than 1600, containing between 2 and 300000 of the sick.

In the course of the following century, it began, without any very obvious cause, to diminish in frequency as well as in virulence; and it ceased almost altogether towards the close of the fifteenth century.

The Scurvy then took the place of the Lepra; and its epidemic march is a very striking example of the metamorphosis of the general constitution of European health. The German nations seemed to have been most alarmed at the outbreak of this new pestilence: its appearance coincided with that of the *sweating-sickness* (la suette Anglaise) in the army of Henry VII. in 1486.

From this period, the scurvy became a prevailing *dyscrasis*, which affected the type or character of almost all other diseases; thus rendering what was comparatively mild and innocuous of a most alarming import. Without mentioning its frequency among sailors, we may state that it was one of the most destructive scourges in the armies of almost every European nation. During the last sixty or eighty years it has been, comparatively, but little known.

Contemporaneously with scurvy, Typhus fever has been one of the most widely-spread epidemics throughout Europe, for the last three centuries.

These two diseases, the one of a chronic or apyretic, and the other of an acute or febrile character, characterise in an especial manner the *general dyscrasis* of the European nations since about the year 1480.

A very peculiar feature of the Scurvy is the circumstance of its blending or allying itself, as it were, with other diseases, and thus modifying and aggravating their usual character or type.—*It was an alliance of this sort*, says our author, *that engendered the disease of syphilis at the close of the fifteenth century*.

Various conjectures as to the origin of this new pestilence, Syphilis, have been formed at different times; some tracing it from America, others from the army of Charles VIII. in Italy, &c. But all these hypotheses, supported although they have been with much elaborate erudition, are equally incorrect. It might be, with quite as much plausibility, derived from England, or from Germany, or from Egypt; for its primitive or essential forms existed in every country for ages before the close of the fifteenth century; and the new and alarming symptoms, which it displayed in 1495, are to be attributed altogether to its being complicated with the scorbutic or putrid diathesis then existing.

Syphilis is therefore to be considered as an old and well-known disease—aggravated at a certain epoch by the addition of a new morbid element. If we follow its career during the last three centuries, we shall find that it has been always alarming, just in proportion as it was allied or complicated with scurvy and the petechial form of typhus fever.*

During the last fifty or sixty years, the Scorbutic diathesis having almost entirely ceased to exist in Europe, the Syphilitic disease has resumed its original or primitive conditions.

This amelioration is attributable, therefore, not so much to the remedies which have been employed, or to any improved method of treating the disease, as to a spontaneous and natural change in its essential elements.

Following the historical succession of Morbid Diatheses, which have presided over the countries of Europe, the *Scrofulous* may be said to succeed to the *Scorbutic;* less terrible in its outward features, but quite as destructive in its ravages as its predecessor. When we consider that the tuberculous *dyscrasis* is only one of the forms of Scrofula, and that this is the parent source of pulmonary consumption, we can at once appreciate its truly formidable character. Its development may be traced back to the commencement of the seventeenth century—at which period the Scrofulous Disease of the spine appears to have been remarkably prevalent. The evil was much encouraged by the mode of life too common in large towns; the people being crowded together in small ill-ventilated houses, and so many of them engaged at the same time in sedentary unhealthy occupations. Scrofula may be considered as truly and essentially a disease of town-life. It is fostered by whatever has a tendency to enfeeble and enervate the vital energies; and, on the other hand, the tendency to its development is most surely counteracted by the enjoyment of pure air, and of active and even laborious exercise.

Such is the *ensemble,* says Dr. Hecker, of some of the historical facts connected with the subject of my enquiries, in a general point of view. They exhibit to us an uninterrupted succession of different morbid *diatheses* or *dyscrases,* which have prevailed in Europe at different epochs—without, however, at all revealing anything of their special cause or origin. And yet who can, with any semblance of reason, attribute this series of phenomena to a blind chance, or a mere hap-hazard of Nature?

It is much wiser to confess our ignorance of this curious branch of historical research—most deserving of future investigation—while we, at the same time, recognize the sufficient evidence to convince us that the great *phases,* so to speak, *of national health are obedient to certain laws.—Revue Medicale.*

---

### On the Microscopical Examination of the Urine.

In one of the recent numbers of this Journal (Medico-Chirurgical Review) we gave an analysis of M. Donné's investigation of the urine, when there is an admixture of seminal fluid in it, by means of the miscroscope.

More recently the attention of M. Rayer, the distinguished physician of La Charité, has been directed to a similar train of research; and we are informed by one of his pupils M. Vigla (who has communicated a very interesting memoir to the French Journal L'Experience for Dec. 1837), that already some highly useful discoveries in the Pathology of the urinary secretion have been obtained by his preceptor.

It is, perhaps, scarcely necessary to say, that when the urine is healthy and quite transparent, the microscope can afford little or no information as to its quality or its contents. It is only when either it is charged with an unusual

---

* This view of the history of syphilis will probably be quite novel to most of our readers. It deserves attention, considering the abilities of its propounder, Dr. Hecker; but whether it is likely to be adopted generally, seems to us more than doubtful.

quantity of saline matter, this having the tendency too to deposition, or when it is more or less loaded with foreign animal secretions, such as pus, semen, fatty matter, &c. that we can expect to derive much assistance from any optical experiments.

M. Vigla has entered very minutely into the general investigation of the subject, and has described with much accuracy the phenomena, which he has detected in the urine from an admixture of various substances, such as pus, mucus, albumen, blood, &c. &c.

As a specimen of his researches, we shall extract his notice of the appearances presented by the presence of *fatty matter in the urine.*

"When this sort of urine is allowed to remain at rest for several hours, there appears on its surface a thin pellicle of greasy or oily-looking matter (the *cremor* of semeiologists). To this pellicle are usually attached, in a confused manner, globules of mucus, or crystals of uric acid or of the ammoniaco-magnesian phosphate.

If a minute quantity of this *cremor* be placed on a watch-glass, and a drop or two of æther be poured upon it, we find, upon examination with the microscope, that the globules of mucus and also the crystals of saline matter become much more distinct than before, and that at the same time there appear around the minute mass traces, not equivocal, of the greasy or oleaginous substance, remaining after the evaporation of the æther.

It is not uncommon to meet with globules of fatty matter in the urine, especially when this fluid is albuminous or purulent. These globules, of very unequal sizes, have a peculiar appearance, and are moreover soluble in æther.

Lastly the urine, which is usually called milky and chylous, is found to contain a considerable quantity of fatty matter."

The following extract too will be read with interest.

" 'Dr. Prout states that, in the urine of diabetic patients, he has met with a fluid of the colour of milk, exactly similar to chyle, and which settle slowly to the bottom of the vessel. In such cases, the vinous fermentation goes on very rapidly, the chylous matter seeming to act in the manner of a ferment or yeast.'

We have observed similar phenomena in the urine of some diabetic patients.

The sediment formed a thin layer of a whitish matter, soft and unctuous to the touch: treated with alcohol, it yielded a very minute quantity of oleaginous matter. Examined with the microscope, it exhibited myriads of regular formed globules, transparent, and smaller than those of milk. This urine passed very quickly into a state of vinous fermentation. M. Quevenne, unacquainted at the time with the writings of Dr. Prout, had announced that this matter was not of the nature of chyle, but that it was truly *un ferment aussi actif que celui de biere et peutetre même identique avec ce dernier.*"

M. Vigla proceeds to give an interesting amount of the detection of a case of *simulation*, on the part of a woman who was in the habit of mixing a small quantity of milk with her urine. It was opaque, of a turbid appearance, and altogether like very purulent urine, when first voided.

After remaining quiet for some time, there was deposited a whitish layer, which still much resembled a purulent admixture, that had fallen to the bottom of the vessel: above, the urine was perfectly clear.

On examining this urine with a microscope, M. Vigla was surprised to find, instead of globules of purulent matter, globules much smaller than these, and very similar to those observed in milk. Suspicion was thus awakened; and ultimately the fraud was detected by finding that the urine drawn off by a catheter was perfectly limpid and free from any admixture.

It may be worth while noticing that acetic acid coagulates *caseum* in urine. The phenomenon of coagulation is very obvious under the microscope.—*L'experience.*

---

### On the Relations, in point of numbers, of the Male and Female Sexes.

The object of the author, M. Girou de Buzareingues, is to prove that, *whatever tends to increase the muscular energy of the two parents contributes, by its influence on the organization, to the pro-creation of the male sex.*

(It is impossible to give an analysis of this curious paper, in consequence of the number of arithmetical tables appended to it. All therefore which we can do is to cull a few,—detached and mutilated though they be,—extracts here and there.)

He alludes to former works in which he has shewn that the above position holds true in all ranks and stations of life. The number of the births of girls, he says, is almost always inferior in the marriages of young than of aged parents; while the reverse is the case in the first fruits of such marriages as take place in seasons of idleness, dissipation and debauchery—the number of female infants being, under such circumstances, very generally greater than that of male infants.

In reference to the season of the year, more marriages, it seems, take place throughout France in January and February than in any other two months.

The first children are usually born in the tenth or eleventh month after marriage, and consequently within one year of its celebration.

The second birth commonly occurs in from twelve to fifteen months after the first.

In general, there is a greater proportional number of female than of male infants in first births; and, on the other hand, the number of male births is usually higher than that of the female, in second conceptions.

The relative number of male births was very high in the year 1811. This might have been owing partly to the numerical decrease in the number of marriages that year, and partly to the national movement occasioned by the marriage of Napoleon, (mouvement national de 1810, occasionné par le mariage de l'Empereur)!

It is also ascertained that the relative number of male births was high in the year 1815, in consequence of the *movement* of the preceding year; and that the same holds true of the years 1808, 9, 10, 11, and 12, owing, no doubt, to the activity and excited energies of the whole nation.

On the other hand, the three or four years preceding the last expulsion of the Bourbons were years of repose, and we know that the relative number of female births was high during this period.

We have already said that in general there is a greater number of female births among the more aged than among young married people. But this proportion varies a good deal in different parts of the kingdom.

An influence, which seems to be much more potent in determining the proportionate number of births of the two sexes, is the condition of the country in respect of peace and ease on the one hand, or of excitement and national bustle on the other.

The truth of this fact was exemplified at several epochs of the Imperial sovereignty; as it is now found that the years of highest prosperity and abundance were always followed by a relative increase of female births, and *vice versa.*

It is a remarkable fact that the maximum of female births during the reign of Napoleon was in the prosperous years of 1806 and 1810; and it is equally deserving of notice that, during the four consecutive years of 1813, 14, 15, and 16, the relative number of female births remained steadily below the usual medium or standard.

The same inference is deducible from an inspection of the general tables of births throughout France during the reign of the Emperor, and also during the period of the restored sovereignty.

From these it appears that the proportional number of the births of the male sex was considerably greater during the former than during the latter period of time.*—*Revue Medicale.*

---

* Most of our readers are probably aware that the actual number of male

*Remarks.*—The observations of our author seem to us to be more droll and amusing than instructive.

We are much inclined to believe that it is not given to us to know anything of the causes which influence the generation of the two sexes, in relation to the comparative frequency of each other. This is one of the mysteries, which we may examine in reference to *statistics,* but which is probably not to be explained on any *physiological* hypothesis.

Indeed some of the data, brought forward by M. Girou, seem to us to argue against the very conclusions which he has drawn from them.—*Rev.*

---

### MEDICO-LEGAL CONSIDERATIONS ON THE MOST FREQUENT CAUSES OF SUDDEN DEATH, AND MORE ESPECIALLY ON ONE HITHERTO VERY LITTLE KNOWN—THE DISENGAGEMENT OF A GASEOUS FLUID IN THE BLOOD.

The author of the following observations is M. Ollivier, of Angers, a member of the Royal Academy of Medicine, who has of late years very successfully devoted himself to the advancement of medico-legal science.

He commences his memoir by specifying the most frequent causes of sudden death, classifying them according to the organs in which they have their seat.

I. *Lesions of the Encephalon.*

He adduces three cases—one of *cerebral hæmorrhage,* another of *apoplexy in the medulla oblongata,* and a third of *purulent meningitis.*

In the *first* of these cases, the examination of the body did not take place until three months after interment. The husband of the patient had been suspected of having caused her death; but the appearances on dissection—the chief of which was *une alteration particuliere resultant de la decomposition putride*—satisfied M. Ollivier that she had died from an extensive cerebral hæmorrhage in the right anterior lobe of the brain.—*Archiv. Gener.* p. 467. 1831.

In the *second* case—that of Apoplexy in the medulla oblongata, which occurred in a healthy old man—the death was almost instantaneous.*

The *third* case is interesting, as illustrative of what extensive organic disease may exist, while the patient is still able to attend to his ordinary duties.

A labouring mason went to his work near the Hôtel de Ville, at the usual early hour in the morning. He soon however, began to complain of great debility; and this so much increased in a short time that he was obliged to return home. He died in the course of the evening.

On dissection, a puriform exsudation was found in the sub-arachnoid cellular tissue, diffused over the greater part of the upper surface of the cerebral hemispheres.

There was no trace of any sanguineous effusion; and the thoracic and abdominal viscera were apparently quite sound.

M. Ollivier alludes *en passant* to two other cases of sudden and unexpected death from cerebral Meningitis.

II. *Lesions of the Lungs.*

Cases of sudden death from this cause

---

births almost always and everywhere exceeds that of female births, in the ratio of 1000 to from 935 to 950 or 60.

---

* M. Ollivier, in alluding to the great frequency of sudden death from cerebral apoplexy, adds that 'the well-known symptoms of this disease cannot generally be mistaken.'

As a general position, the truth of this statement cannot be gainsayed; but it is more than doubtful that many cases of *very sudden and instantaneous death* are owing to apoplexy of the cerebrum, unless indeed the medulla oblongata be at the same time implicated: it is, however, rather to the *heart* than to the *encephalon* that we are to look for the cause of the disease under such circumstances.

are of less frequent occurrence than from the former. M. Ollivier however reports two very remarkable cases, which well deserve notice.

4. *Emphysema of the Lungs.* Two men from quarrelling came to blows. They were however speedily separated. The stronger and older, being obliged by the interference of the attendants to master his resentment, hurried directly home; but no sooner had he reached the door of his house than he dropped down dead.

It was at first suspected that he must have received some blow in the scuffle; and M. Ollivier was therefore ordered by the public authorities to examine the body. *The cause of death,* says he, *was quite natural: it had been occasioned by a spontaneous emphysema of both lungs.**

The experiments of M. Leroy on asphyxia can leave no doubt that sudden death may be occasioned by this morbid state of the respiratory organs.

5. *Pulmonary Apoplexy.* A middle-aged man, in a violent fit of passion, was in the act of rushing forwards to strike his adversary, when he suddenly became deadly pale, staggered for a step or two, fell down, and expired.

Such, at least, was the story of the other man.

An examination of the body was therefore ordered, and M. Ollivier performed it.

There were no marks of any outward violence on the body, and the only internal lesion to account for the death of the patient was an extensive *pulmonary apoplexy.*

"Both lungs were found to be unusually firm, resisting, and heavy;—when divided, their tissue exhibited a deep blackish red colour; and all the branches of the pulmonary veins and arteries were found filled with clots of blood."

M. Ollivier remarks that certain communications, which have been made to him from the Bicetre hospital, have led him to believe that Pulmonary Apoplexy, or *engouement* (congestion), is an occasional cause of sudden and unexpected death in old people.

6. *Double Pleuro-pneumonia.*—A young woman, five months advanced in pregnancy, had been ailing for a few days; but this had not prevented her from attending to her domestic duties. She was preparing to go to her sister's house, when, on the return of her servant in the course of half-an-hour afterwards, she was found dead on the floor.

The dissection proved that *la mort avait été causée par une pleuro-pneumonie double.*

Such an occurrence is rare in early life; but is not unfrequently met with among the aged inmates of such hospices as the Bicetre, Saltpetriere, &c.*

### III. *Lesions of the Heart and great Blood-vessels.*

From the researches of M. Ollivier, (recorded in the article *Ruptures of the Heart, &c.* in the 8th vol. of the Dictionnaire de Medecine,) it follows—and his experience no doubt quite agrees with that of other pathologists—that most cases of *instantaneous* or of *very sudden death* are attributable to some form of this dreadful lesion. It also appears that, in not a few cases of this description, there has been, previously to the fatal attack, an almost entire absence of any cardiac distress; and hence that the real cause of death has not been even suspected before the dissection of the body.†

---

* The curious reader will find other similar cases in the recent works of MM. Piedagnel, Pillore, and Leroy, alluded to by our author.

* In the Foreign Periscopic Department of one of the recent numbers of this Review, our readers will find an interesting memoir on the frequency of sudden death among the aged inmates of such establishments from *unsuspected* pneumonia: in several of the cases the patients had been seemingly in their ordinary health a few hours before their decease.—*Rev.*

† We cannot too urgently impress attention to this melancholy truth on the minds of medical men.

*Case 7. Rupture of the Pulmonary Artery.* A healthy robust youth, 22 years of age, was in a scuffle wounded in the neck; he fell down on the spot, and died almost directly. Very little blood flowed from the wound. On dissection the wound, situated about an inch above and to the outside of the sternal extremity of the right clavicle, was found to have penetrated two inches in depth, and to have passed between the trachea and the left subclavian vein; but neither had these important organs suffered any injury, nor were any other considerable blood-vessels wounded.

On opening the cavity of the chest, nearly *five pounds* of black coagulated blood were found in the left bag of the pleura; the cavity of the pericardium also contained a quantity of clotted blood. This sero-fibrous envelope exhibited an irregular rupture, nearly two inches in length, at the point corresponding to the root of the left lung: the pulmonary tissue also there was infiltrated with dark blood. The pulmonary artery was found to have given way, just beneath the point where the pericardium is reflected upon this vessel: its parietes did not however exhibit any traces of morbid change.—*Archives Générales.*

We shall conclude this memoir in our next number, and we shall then adduce several cases to prove that sudden death is sometimes owing to the extrication of a gaseous fluid in the blood itself.—*Rev.*

---

It is an awful, but a salutary, admonition to avoid rashness and over-confidence in the pursuit of our profession.

USEFUL APPLICATION TO BED-SORES.

A correspondent of the Bulletin General de Therapeutique recalls to the attention of medical men a very excellent, and easily prepared, local application for those troublesome and distressing sores which are so apt to occur in bed-ridden patients.

It is unnecessary to allude to the frequency of this annoyance in certain cases of protracted disease, more especially of obstinate fevers, of phthisis, &c., and to the extreme difficulty of counteracting it. The late M. Autenrieth of Vienna was much in the habit of using the thick sedimentary deposit obtained by adding the liquor plumbi, drop by drop, to a strong decoction of oak-bark—in short a *tannate of lead*—as a topical remedy to bed-sores, with great success. The super-natant liquor being decanted off, the sediment is easily procured; it is then to be spread on linen, as we do with an ointment. The application to the abraded surface should be repeated every night and morning.

Dr. Tott, a countryman of M. Autenrieth, has of late years used this remedy with very satisfactory effects. In some cases, where it did not seem to agree, he mixed a certain portion of the *tannate*, previously dried, with simple ointment, (two drachms to one ounce); and he found that the sores often healed readily under the use of this cerate.——*Bulletin General.*

We can bear our testimony in favour of the good effects of this application for bed-sores.

In our own practice we have prepared it by mixing together the liquor plumbi and the common tincture of kino.—*Rev.*

